# Dual‐Function Piezo‐Photocatalytic Systems for Sustainable Hydrogen Evolution and Environmental Remediation

**DOI:** 10.1002/advs.202513811

**Published:** 2025-10-27

**Authors:** Nguyễn Hoàng Ly, Sang Jun Son, Hesam Kamyab, Yasser Vasseghian, Sang‐Woo Joo

**Affiliations:** ^1^ Department of Chemistry Gachon University Seongnam 13120 South Korea; ^2^ Department of Biomaterials Saveetha Dental College and Hospital Saveetha Institute of Medical and Technical Sciences Chennai 600077 India; ^3^ UTE University Faculty of Architecture and Urbanism Architecture Department Research Group TCEMC 170527 Quito Ecuador; ^4^ Department of Chemistry Soongsil University Seoul 06978 South Korea; ^5^ Centre for Herbal Pharmacology and Environmental Sustainability Chettinad Hospital and Research Institute Chettinad Academy of Research and Education Kelambakkam Tamil Nadu 603103 India

**Keywords:** CO_2_ reduction, Hydrogen evolution, organic pollutants, Piezo‐photocatalytic

## Abstract

Hydrogen (H_2_) production and environmental cleanup, including pollutant breakdown, nano‐plastic removal, and CO_2_ reduction, are crucial for achieving environmental sustainability. Piezo‐photocatalysis has appeared in an optimistic approach to address environmental pollution and the escalating energy crisis. Although several reviews on H_2_ production and environmental cleanup using piezo‐catalytic technologies have been recently published, there is no review specifically focused on the literature related to dual‐functional piezo‐photocatalytic systems. This research aims to fill that gap as the field continues to grow rapidly. This study reviews dual‐function piezo‐photocatalytic systems, which can be easily fabricated to enhance the effective uncoupling and transfer of photoproduced holes and electrons for H_2_ production and environmental cleanup. First, piezoelectric materials, such as metal oxides (e.g., TiO_2_, ZnO, BaTiO_3_), 2D materials (e.g., MoS_2_, MXenes, graphene‐based materials), perovskite materials, and composite/heterostructure materials, are introduced. Second, this work also explores various modification methods that enhance piezo‐photocatalytic efficiency, highlighting the remarkable properties of dual‐function systems designed for sustainable H_2_ production and environmental cleanup. Additionally, this work provides insight into the underlying mechanisms of piezo‐photocatalytic activity and suggests new pathways toward high‐performance piezo‐photocatalysts. Finally, this research discusses future directions for piezoelectric materials in environmental applications and sustainable H_2_ production.

## Graphical abstract

This article reviews dual‐function piezo‐photocatalytic systems that combine light and mechanical energy to produce hydrogen and clean the environment. It explains how various materials, including metal oxides, 2D structures, perovskites, and composites, enhance charge separation and reaction efficiency. The study highlights their role in pollutant degradation, CO_2_ reduction, and hydrogen evolution.

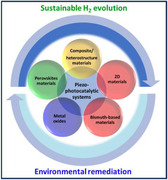

## 1 Introduction

Concurrent H_2_ generation combined with environmental remediation—such as nano‐plastic degradation,^[^ ^1^ ^]^ pollutant breakdown,^[^ ^2^ ^]^ CO_2_ conversion,^[^ ^3^ ^]^ and others—is an attractive approach to tackling environmental and energy challenges in sustainable development.^[^ ^4^ ^]^ In traditional photocatalytic H_2_ production, the reducing ability and mechanism of photo‐excited electrons have garnered increasing attention, especially regarding the oxidation process involving the role of holes.^[^ ^5^ ^]^ Usually, the activity of sacrificial holes is terminated using toxic CH_3_OH organic compounds or inorganic sulfides. However, using supplementary sacrificial agents raises the price of H_2_ production and increases the peril of generating harmful toxicants, which is inadmissible for workable purpose.^[^ ^6^ ^]^ Organic pollutants in environmental water could serve as a solution rather than a problem. The availability of cost‐free sacrificial agents offers a new way to enhance photocatalytic H_2_ production via water splitting. Replacing conventional sacrificial reagents with chemical pollutants to boost H_2_ generation is an inspiring and innovative procedure.^[^ ^7^ ^]^ Wastewater organic pollutants can serve as hole sacrificial species for H_2_ production; however, studies indicate that not all pollutants are equally effective. For efficient H_2_ production, the photocatalyst must generate •OH radicals from photogenerated positive holes, and the pollutants should be easily degraded by these radicals. Therefore, there is a vital need to design a photocatalytic system that effectively performs environmental cleanup while also producing H_2_. Notably, the dual‐function piezo‐photocatalytic system shows high H_2_ output, highlighting its potential for efficient H_2_ generation from wastewater and simultaneous environmental cleanup, such as degrading multiple pollutants. Many researchers have explored metal–organic frameworks,^[^ ^8^ ^]^ single‐atom catalysts,^[^ ^9^ ^]^ hybrid piezo‐photocatalysts,^[^ ^10^ ^]^ piezo‐catalysis and piezo‐photocatalysis,^[^ ^11^ ^]^ boosting photocatalytic H_2_ evolution via piezo‐stimulated polarization,^[^ ^12^ ^]^ and related techniques.

By converting mechanical energy from external stimuli (e.g., vibrations, friction, wind, tides, etc.), piezoelectric catalysts have shown appreciable promise for green H_2_ energy generation and pollutant degradation.^[^ ^13^ ^]^ Piezo‐photocatalyst materials are classified into several categories according to their composition/morphology: metal oxides (e.g., ZnO,^[^ ^14^ ^]^ BaTiO_3_,^[^ ^15^ ^]^ BiFeO_3_,^[^ ^16^ ^]^ etc.), 2D materials of transition metal dichalcogenides,^[^ ^17^ ^]^ carbon nitride sheets,^[^ ^18^ ^]^ and O‐doped MoS_2_,^[^ ^19^ ^]^, perovskite materials (e.g., Bi_2_WO_6_,^[^ ^20^ ^]^ CH_3_NH_3_PbI_3_,^[^ ^21^ ^]^ acidified La_2_NiO_4_,^[^ ^22^ ^]^ etc.), and composite/heterostructure materials (e.g., ZnO/ZnS/MoS_2_,^[^ ^7^ ^]^ Bi_4_Ti_3_O_12_@C,^[^ ^23^ ^]^ BiOBr/BaTiO_3_,^[^ ^24^ ^]^ ZnIn_2_S_4_‐BiOCl,^[^ ^25^ ^]^ etc.). Various piezo‐photocatalytic materials have been well‐controlled in shape, including 0‐, 1‐, 2‐, and 3D structures (e.g., 5%‐Cl‐ZnO nanorods,^[^ ^26^ ^]^ Cd_x_Zn_1‐x_S solid‐solution 1D nanorods,^[^ ^27^ ^]^ 2D CuInP_2_S_6_,^[^ ^28^ ^]^ 2D/1D g‐C_3_N_5_/Bi_8_(CrO_4_)O_11_,^[^ ^29^ ^]^ etc.). Especially, to improve electromagnetic absorption and reduce the recombination of charge carriers that take a critical part in enhancing photoconversion efficiency, composite/heterostructure materials have been developed exhibiting hollow interiors.^[^ ^30^ ^]^ In addition, numerous materials have been investigated with special morphologies suitable for a specific purpose, such as organic dye degradation using nanofibers,^[^ ^2^ ^]^ photocatalytic H_2_ evolution via CuS@NaNbO_3_ nanorods,^[^ ^31^ ^]^ Sn_3_O_4_ nanoflowers/Mn_0.5_Cd_0.5_S for H_2_ evolution,^[^ ^32^ ^]^ piezo‐catalytic CO_2_ reduction using Au@BaTiO_3_ yolk‐in‐shell nanostructure,^[^ ^33^ ^]^ degradation of pollutants utilizing BaTiO_3_@ReS_2_ Schottky,^[^ ^34^ ^]^ etc.

**Figure 1:**
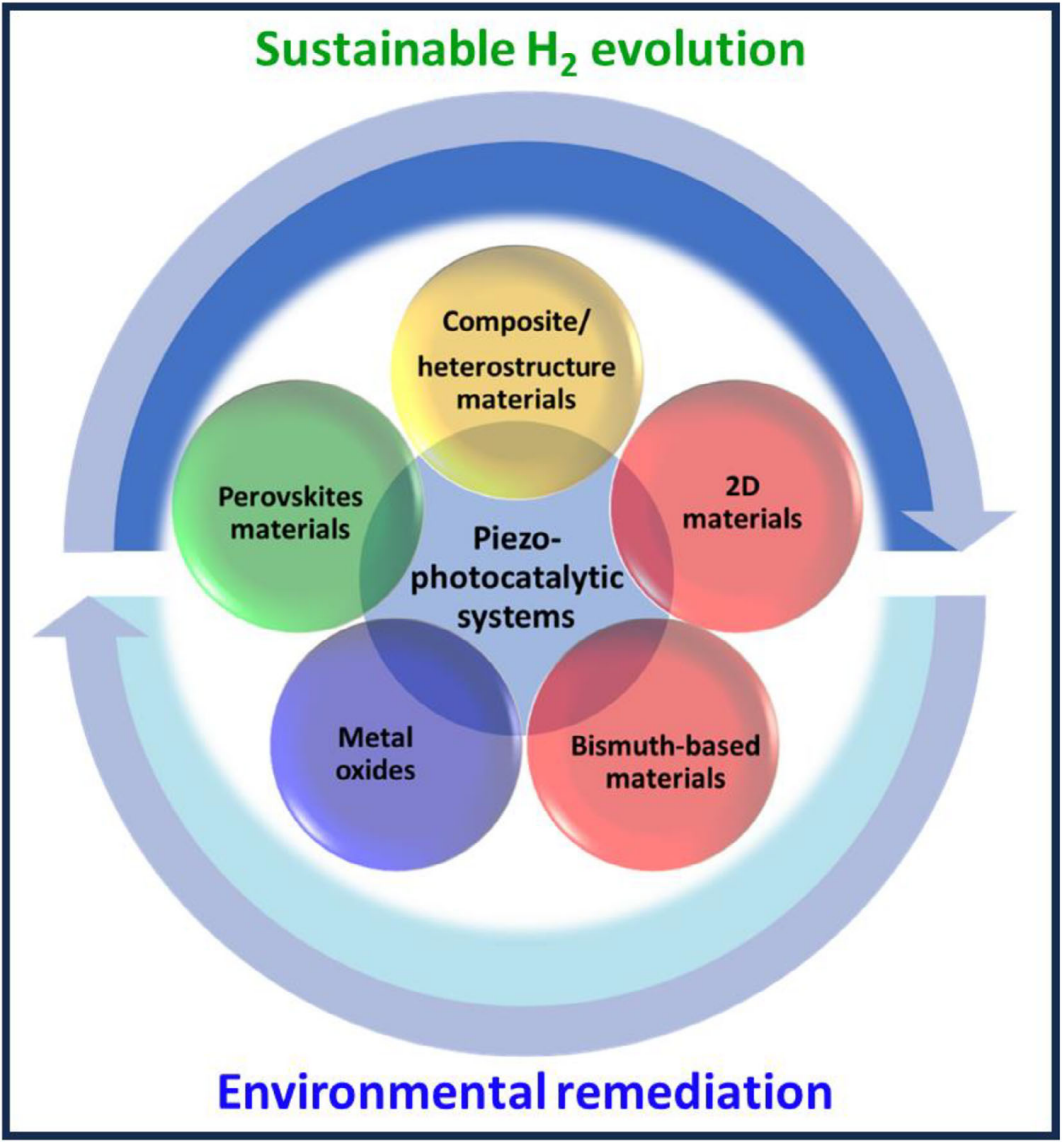
Various materials are employed for piezo‐photocatalytic systems for the H_2_ evolution and environmental remediation.

Recently, several reviews have been published on piezo‐photocatalytic H_2_ evolution and wastewater treatment using piezo‐photocatalysts,^[^ ^13^ ^]^ environmental remediation with piezo‐catalytic technologies,^[^ ^35^ ^]^ water treatment through piezo‐catalysis,^[^ ^36^ ^]^ energy and environmental applications via high‐performance piezo‐photocatalytic heterojunction systems,^[^ ^37^ ^]^ and reducing harmful environmental pollutants with graphene‐based catalysts,^[^ ^38^ ^]^ but there is no review focusing on the literature on dual‐functional piezo‐photocatalytic systems. This is the gap that this work aims to fill, as the field is rapidly evolving. This study concentrates on the dual and multifunctional uses of piezo‐material‐derived photocatalysts as innovative solutions for pollution control and hydrogen energy production—areas that have not been fully explored in past reports, while considering future prospects. The authors systematically examine piezo‐photocatalytic materials that exhibit unique physicochemical and piezoelectric properties, with the goal of using these systems for clean H_2_ energy generation and environmental applications (**Figure** **1**). The studies demonstrate that the improved catalytic property is attributed to the light‐ and ultrasound‐generated piezoelectric field effects on composite/heterostructure materials. Modulated by a piezoelectric field, the reactions and mechanisms behind H_2_ generation, CO_2_ reducing reaction, and the decomposition of chemical pollutants are explained through the energy band structure of composite/heterostructures and the pathways of electron transfer. The authors anticipate this work will not only facilitate the development of bifunctional piezo‐photocatalytic systems but also serve as valuable guidelines for future applications of composite and heterostructure materials in piezo‐photocatalytic processes. These processes involve simultaneous environmental remediation (such as degradation of contaminants and CO_2_ reduction) and clean H_2_ energy production. Additionally, it will guide those aiming to create the most effective and sustainable systems, address key challenges, and broaden understanding in this emerging field.

## 2 Fundamentals of Piezo‐Photocatalysis

**Figure 2:**
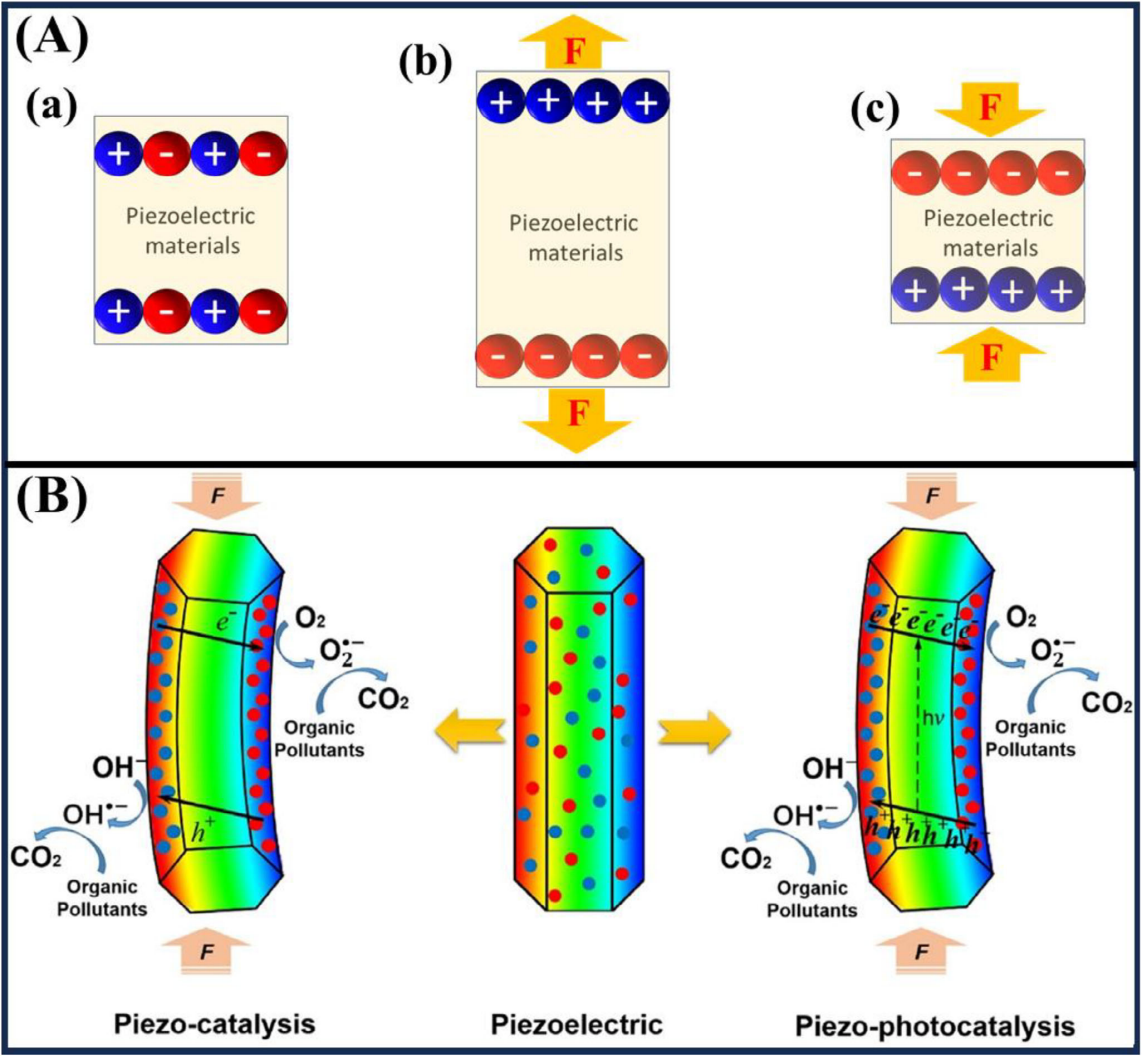
(A) Schematic illustration of the piezoelectric phenomena, including the internal charge of (a) no applied, (b) stretched, and (c) compressed forces) The relationship between piezoelectric, piezo‐catalysis, and piezo‐photocatalysis.^[^ ^46^ ^]^ Reproduced with permission.^[^ ^46^ ^]^ Copyright 2019, Elsevier.

The term “piezo” comes from the Greek “piezein,” meaning “to press” or “pressure.” This aligns well with its application in science, particularly in the field of piezoelectricity. Piezoelectricity was initiated by French physicists Jacques and Pierre Curie in 1880.^[^ ^39^ ^]^ They found that squeezing certain crystals, such as tourmaline, quartz, topaz, and Rochelle salt, generates an electrical charge. They also observed that these crystals change shape when an electrical current is applied, indicating the process is reversible (as shown in **Figure** **2**A). This finding laid the groundwork for the production of many piezoelectric components. Historically, advances in science and materials technology have led to the development of numerous types of piezoelectric materials such as single crystallites, ceramics, and thin films. These materials generate electricity from vibrations that would otherwise be wasted, making them highly valuable for energy harvesting. Piezoelectric materials are generally classified into two categories: natural materials and artificial synthetic materials. Natural materials are dielectric crystals that are anisotropic and have a non‐centrosymmetric crystal lattice. Examples include quartz, minerals, etc. Manufactured materials are synthetic ferroelectric substances used in piezoelectric applications for the energy crisis and environmental pollution.^[^ ^40^ ^]^ They can be grouped into five types: quartz‐like analogs, ceramics, polymers, composites, and thin films.

Piezoelectric materials exhibit characteristics of polarization or alterations in their polarization state when subjected to variations in force or electric fields. These materials serve as intermediaries between mechanical energy and chemical energy, transforming such stress conditions into chemical energy.^[^ ^41^ ^]^ Toxic organic pollutants present in wastewater from factories, treated with this chemical energy, can aid in breaking down the toxins in natural waterbodies.^[^ ^24^ ^]^ Remarkably, one of the most important physical properties of the piezoelectric materials is their dielectric constant.^[^ ^42^ ^]^ In general, a higher dielectric constant means that, under the same strength of electric field or force field, a higher degree of polarization is induced. Meanwhile, polarization dielectric has three polarization types of electron displacement, ion displacement, and orientation.^[^ ^12^ ^]^ Most classical piezoelectric materials (especially perovskites) possess non‐integrated positions of negative and positive charge centers inside the ions/atoms, and therefore experience an electron displacement polarization.^[^ ^43^ ^]^ Source of piezoelectricity the most significant phenomenon related to piezoelectric materials is the piezoelectric effect. This happens because when a piezoelectric crystal is strained due to an externally acting force, an electrical charge builds up on the face of the material that is linearly dependent on the applied force.^[^ ^44^ ^]^ By studying crystallography, researchers can determine which crystals will likely be piezoelectric.^[^ ^45^ ^]^ An important case is crystals, which do not have a center of symmetry, resulting in piezoelectricity. In response, the crystal lattice bends under pressure, separating positively and negatively charged particles. This distortion induces a dipole moment in each molecule.^[^ ^17^ ^]^

Schematic diagrams of piezo‐catalysis, piezoelectric, and piezo‐photocatalysis are shown in Figure 2B. The charge pairs generated by photoexcitation of a photocatalyst may recombine due to its bandgap. In piezo‐catalysis, mechanical stress can induce charge separation in the material, which can occur under external conditions such as ultrasonic waves. Piezo‐photocatalysis enhances this process: when mechanical vibrations and light are out of phase, it produces a more effective electron–hole pair, leading to increased electron transfer to the surface or lowering the energy level of the bonded polar material.

Figure 2B describes the piezo‐photocatalysis process of the piezoelectric materials for organic contaminant degradation and contrasts it to the piezo‐catalysis process.^[^ ^46^ ^]^ In piezo‐photocatalysis, applying mechanical stress deforms a piezoelectric material, generating a built‐in electric field called the piezo‐potential. This field helps separate light‐excited electron‐hole pairs into the conduction and valence bands. As a result, it reduces recombination and extends the existence period of electrons and holes, increasing the photocatalyst's efficiency. The piezo‐potential acts as a guiding force, directing these mobile charges to one side tojoin in redox reactions. Accordingly, the heterostructure composites exhibit better photodegradation performance compared to the mono‐junction. Force and light generate polarization field in the piezoelectric materials will drive the photoelectrons and photo‐holes separation, subsequently enhancing the separation of photo‐holes and photoelectrons in the piezo‐photocatalysis.^[^ ^11^ ^]^ This makes them migrate in opposite directions toward the piezoelectric materials surface for reduction‐oxidation reactions. In addition, the production of ^•^O_2_^−^ and ^•^OH reactive species with respect to the redox potential values of O_2_/ ^•^O_2_^−^ at −0.33 V, H_2_O/^•^OH at +2.38 V, and OH^−^/ ^•^OH at +1.99 V versus normal hydrogen electrode leading to more organic pollutants are degraded in piezo‐photocatalysis than in single either piezo‐catalysis or photocatalysis.^[^ ^47^ ^]^

Although extensive studies have been conducted on homo‐ and hetero‐catalysts separately, their combined use for multifunctional applications—especially in solving complex problems like water pollution, sustainable energy, and climate change—has recently gained attention.^[^ ^48^ ^]^ The advantage of dual‐functional piezo‐photocatalysis mainly comes from its improved catalytic performance, which results from the synergistic combination of piezoelectricity and photocatalysis.^[^ ^49^ ^]^ This not only helps prevent charge carrier recombination but also enhances pollutant degradation and other chemical reactions. By integrating catalysis with both mechanical activation (such as ultrasound) and light activation, this dual‐function approach significantly boosts efficiency and reduces external power needs.^[^ ^50^ ^]^ As a result, this method simplifies system design and lowers costs. However, developing a composite piezo‐photocatalyst capable of tackling both issues remain a major challenge. The section “**8. Dual‐function piezo‐photocatalytic systems**” highlights recent research on dual‐functional piezo‐photocatalytic reactions. In addition, **Table** **1** shows more advantages and disadvantages of piezo‐photocatalysts and traditional photocatalysts.

**Table 1:** Comparison of piezo‐ and traditional photocatalysts.^[^ ^5^, ^7^ ^]^

	Piezo‐photocatalysts	Traditional photocatalysts
**Activation mechanism**	✓ Light energy ✓ Mechanical stress	✓ Light energy
**Charge separation**	✓ Hinder the charge recombination ✓ Promote the reaction rates ✓ Enhanced catalytic performance	✓ Face constraints in the recombination of charge ✓ Lower overall efficiency
**Performance under varied conditions**	✓ Mechanical stress (e.g., vibrations) alongside available light ✓ Natural light	✓ Specific light wavelengths ✓ Intensities
**Application scope**	✓ Pollution degradation ✓ Energy conversion ✓ Synthesis processes ✓ CO_2_ reduction	✓ Water purification ✓ Air treatment ✓ Photocatalytic CO_2_ reduction ✓ Pollution degradation ✓ Energy conversion ✓ Synthesis processes
**Environmental impact**	✓ Greater energy efficiency ✓ Sustainability	✓ Specific energy sources (e.g., UV light)

## 3 Materials for Piezo‐Photocatalytic Systems

### 3.1 Metal Oxides

Metal oxide nanostructures (e.g., TiO_2_, ZnO, BaTiO_3_, etc.) have also been used to increase the β‐phase content of polymers, leading to a significant enhancement of piezoelectric activity.^[^ ^51^ ^]^ This enhancement is attributed to the cubic closest‐packed configuration of these nanoparticles and their anomalously elevated surface energy, which suppresses changeable thermodynamic phases. The presence of non‐native crystal phases and asymmetry in such metal oxides further renovates the physicochemical characteristics through compounding. Magnetoelectric polymeric mixture may provide superior piezoelectric output potential. For example, within 24 min, only under ultrasonic vibration, V‐doped SrTiO_3_ nanofibers exhibited excellent piezo‐catalytic performance of bisphenol A removal ≈ 100%.^[^ ^52^ ^]^ Also, BiFeO_3_/Pd achieved a high yield of 11.4 µmol·h^−1^ H_2_ evolution_3_.^[^ ^16^ ^]^ Interestingly, under ultrasonic vibration, piezoelectric BaTiO_3_ nanowires exhibit enhanced effective piezo‐catalytic performance more than BaTiO_3_ nanoparticles.^[^ ^53^ ^]^ Besides, piezoelectric photocatalytic tetragonal BaTiO_3_ nano‐cubes exhibited the highest performance of CO_2_ conversion to CO with a rate of 52.9 mmol·g^−1^·h^−1^.^[^ ^54^ ^]^ A core–shell Z‐scheme heterostructure, formed by BaTiO_3_ nanowires covalently bonded with an imine‐linked covalent organic framework, demonstrates an impressive yield of 33 mmol·g^−1^·h^−1^ of H_2_ evolution.^[^ ^55^ ^]^ Furthermore, piezo‐photocatalytic features of other materials are listed in **Table** **2**.

**Table 2:** Summary on hydrogen evolution and environmental remediation by piezoelectric materials.

	Catalyst	Scavenger	Light/ Vibration	Application	Performance	Reference
**Metal oxides**	P123@SrTiO_3_	TEOA	300 W Xe lamp	H_2_ generation	402 µmol·g^−1^·h^−1^	[83]
	BiFeO_3_	Na_2_SO_3_	Ultrasonic vibration	H_2_ evolution	11.4 µmol·h^−1^	[16]
	V‐doped SrTiO_3_		Ultrasonic vibration	Bisphenol A removal	100% within 24 min	[52]
	MoSe_2_ decorating TiO_2_	Na_2_SO_4_	300 W Xe lamp	4‐nitrophenol and Cr^6+^ removal		[84]
	BaTiO_3_		UV light radiation and ultrasonic vibration	MO degradation	≈98.17% within 80 min	[15]
	BaTiO_3−x_		Light and ultrasound	H_2_ evolution	132.4 µmol·g^−1^·h^−1^	[85]
	AgNbO_3_		Light and ultrasound	RhB removal	72.8% within 150 min	[86]
	Quartz/TiO_2_	Lactic acid	Light and ultrasonic	H_2_ evolution	20.4 mmol·g^−1^·h^−1^	[87]
	OH‐modified SrTiO_3_	CH_3_OH	Light and ultrasonic	H_2_ evolution	701.2 µmol·g^−1^·h^−1^	[6]
	Cl‐ZnO	Na_2_SO_4_	Ultrasonic vibration	RhB removal		[26]
**2D materials**	2D g‐C_3_N_4_‐based materials	TEOA	> 420 nm	H_2_ production	5192.2 µmol·g^−1^·h^−1^	[18]
	SrBi_8_Ti_7_O_27_ nanosheets		Solar irradiation	CO_2_ reduction	361.34 µmol·g^−1^·h^−1^	[79]
			Ultrasonic vibration	Degradation of organic pollutants	100% efficiency within 60 min	
	Ti_3_C_2_Tx on ZnO films		Illumination and ultrasonic irradiation	H_2_ evolution		[56]
	Sailboat‐like‐vertical‐MoS_2_‐nanosheets		Ultrasonic/stirring	H_2_ evolution	1598 µmol·g^−1^·h^−1^	[4]
				RhB degradation	94% within 60 min	
	Bi_12_ZnO_20_	MeOH	> 420 nm	H_2_ evolution	18.701 mmol·g^−1^	[88]
				RhB degradation	98.5% within 70 min	
	MoSe_2_ nanosheets		300 W Xe lamp	RhB degradation	94.4% in 40 min	[89]
				MB degradation	95.1% in 50 min	
	ZnO with Ti_3_C_2_T_x_		Visible light	MB degradation	99.9% in 40 min	[90]
	g‐C_3_N_5_/Bi_8_(CrO_4_)O_11_		Visible light	RhB degradation	94.5% in 60 min	[29]
				TCH degradation	93.3 % in 60 min
	O‐doped MoS_2_		Ultrasonic irradiation	H_2_ production	47.75 µmol·g^−1^·h^−1^	[19]
	LaNiO_3_/g‐C_3_N_5_		395 nm light	TC removal	90.0 % in 60 min	[91]
**Perovskites materials**	Bi_2_WO_6_	TEOA	Ultrasonic cleaner	H_2_ production	191.3 µmol·g^−1^·h^−1^	[20]
	CH_3_NH_3_PbI_3_		Ultrasonic and visible irradiation	H_2_ production	23.30 µmol·h^−1^	[21]
	Bi_4_Ti_3_O_12_		Ultrasonic cleaner	Phenol degradation	95% within 100 min irradiation	[63]
	Acidified La_2_NiO_4_	Na_2_SO_4_	Ultrasonic vibration	H_2_ production	1315.44 µmol·g^−1^·h^−1^	[22]
	NaNbO_3_		UV light	RhB degradation	99.21%	[92]
	SrTiO_3_/MoS_x_	TEOA	Solar light irradiation	H_2_ production	40.57 mmol·g^−1^	[93]
**Composite materials**	CdS/CdCO_3_‐CoS_2_	TEOA	300 W Xe lamp	H_2_ evolution	64 867.88 µmol·g^−1^·h^−1^	[78]
			> 420 nm	CO_2_ reduction	654.7 µmol·g^−1^·h^−1^	
	TiO_2_/g‐C_3_N_4_	Lactic acid	Sunlight	H_2_ evolution	43.57 mmol·g^−1^·h^−1^	[94]
			Visible light		2.9 mmol·g^−1^·h^−1^	
	Na_0.5_K_0.5_NbO_3_	Na_2_SO_4_	Ultrasonic vibration	Degradation of organic pollutants	25.16 × 10^−3^ min^−1^	[95]
	NaNbO_3_/WO_3_		Visible light irradiation and ultrasound vibration	RhB degradation	73.7 % within 120 min	[80]
	0.7 BiFeO_3_‐0.3 BaTiO_3_		Light irradiation and low‐frequency vibration	RhB degradation	99% within 60 min	[81]
	Bi_2_WO_6_/Black TiO_2_		Ultrasonication and sunlight	RhB degradation	98.43% in 60 min	[96]
	BiVO_4_‐rGO‐PVDF		400–700 nm	MB degradation	82% in 180 min	[97]
	Sr‐doped Bi_4_O_5_Br_2_/Bi_2_MoO_6_		300 W visible LED lamp	Nitrite removal	91.7 % in 60 min	[98]
				4‐chlorophenol removal	100 % in 60 min	
	Cd_0.4_Zn_0.6_S	Lactic acid	Visible light and ultrasonic vibration	H_2_ evolution	4.45 mmol·g^−1^·h^−1^	[27]
	NaNbO_3_‐Au‐Sn_3_O_4_		Sunlight irradiation	Carbofuran removal	73 % within 120 min	[99]
	Bi_2_S_3_/ZnSn(OH)_6_	Lactic acid	Ultrasonic coupling illumination	H_2_ evolution	336.21 µmol·g^−1^·h^−1^	[100]
	Sn_3_O_4_/Mn_0.5_Cd_0.5_S		Ultrasonic coupling light	H_2_ evolution	21.46 mmol·g^−1^·h^−1^	[32]
	Pt/PbTiO_3_	C_2_H_5_OH	Ultrasonic coupling light	H_2_ evolution	1181.3 µmol·g^−1^·h^−1^	[101]
	Au‐BiOBr		Ultrasonic coupling light	Carbamazepine removal	95.8% within 30 min	[102]
	BaTiO_3_ @MoSe_2_		Ultrasonic coupling light	H_2_ evolution	4533 µmol·g^−1^·h^−1^	[103]
	SrTiO_3_/BaTiO_3_	Na_2_SO_4_	Ultrasonic coupling light	H_2_ evolution	1950.2 µmol·g^−1^·h^−1^	[104]
	Cu_2_Se decorated CdS_0.95_Se_0.05_	Lactic acid	λ ≥ 400 nm	H_2_ evolution	570.7 µmol·h^−1^	[105]
	ZnO/ZnS/MoS_2_		Ultrasonic coupling light	H_2_ evolution	746.56 µmol·g^−1^·h^−1^	[7]
	CuS/NaNbO_3_	Na_2_S and Na_2_SO_3_	Sunlight	H_2_ evolution	1603 µmol·g^−1^·h^−1^	[31]
	MoSe_2_/Se‐decorated CdS	Lactic acid	Light and stirring	H_2_ evolution	59.1 mmol·g^−1^·h^−1^	[106]

**Abbreviation**: Triethanolamine (TEOA); Rhodamine B (RhB); Methylene blue (MB); polyvinylidene fluoride (PVDF); methyl orange (MO); tetracycline hydrochloride (TCH); tetracycline antibiotic (TC).

### 3.2 2D Materials

The piezoelectric phenomena in a conversion from mechanical energy to electrical energy, is highly promising for functional and intelligent electronic devices. To optimize this feature, scientists have focused on the miniaturization of piezoelectric materials. Interestingly, some of the atomically thin 2D materials (e.g., MoS_2_, MXenes, graphene‐based materials, etc.) demonstrate a pronounced piezo response. Recently, the authors illustrated piezoelectric‐driven photocarrier separation in a 2D interfacial Schottky heterojunction, which is constructed by depositing ultrathin laminate flaky Ti_3_C_2_T_x_ on ZnO films.^[^ ^56^ ^]^ In addition, Au_25_(p‐mercaptobenzoic acid)_18_ nano‐clusters modified red g‐C_3_N_4_ nano‐catalysts exhibiting the rate in the reduction of CO_2_ to CO of 111.95 µmol·g^−1^·h^−1^.^[^ ^57^ ^]^ Besides, to improve piezo‐photocatalytic performance, oxygen vacancy‐modified Bi_4_Ti_3_O_12_ nanosheets exhibit an excellent rate constant (K = 0.214 min^−1^) of RhB degradation, which is enhanced 2.2‐fold compared to pristine Bi_4_Ti_3_O_12_ nanosheets.^[^ ^58^ ^]^ Furthermore, for improved efficient piezo‐photocatalytic, PZT (lead zirconate titanate), MgAl, and GO (graphene oxide) are combined to generate a PZT‐MgAl‐GO composite material, which aims to remove hazardous Cr^6+^ ions under sonication and UV light irradiation.^[^ ^59^ ^]^ Also, piezo‐photocatalytic g‐C_3_N_4_ has been successfully used for H_2_O_2_ production from pure water.^[^ ^60^ ^]^

### 3.3 Perovskites Materials

In recent years, halide perovskites have also shown promising piezoelectric and ferroelectric properties comparable to traditional inorganic piezoelectric materials.^[^ ^61^ ^]^ Nevertheless, even with the growing number of halide perovskite materials that have shown remarkable piezoelectric properties, a thorough understanding of the origin of piezoelectricity in halide perovskites remains lacking, in order for practical applications in this field to be achieved. For example, oxygen vacancy‐modified NaNbO_3_ powders exhibit the best degradation efficiency constant K = 131.7 × 10^−3^ min^−1^.^[^ ^62^ ^]^ Also, Bi_2_WO_6_ layered‐perovskites can achieve 1147.8 µmol g^−1^ of hydrogen yield, after 6 h of vibration, and the hydrogen generation with a rate of 191.3 µmol·g^−1^·h^−1^.^[^ ^20^ ^]^ Specially, in hydroiodic acid solution, piezo‐photocatalytic organolead halide perovskite CH_3_NH_3_PbI_3_ exhibit a superior 23.30 µmol·h^−1^ H_2_ evolution upon light and mechanical stimulations, higher than piezo‐catalytic and photocatalytic with a rate of 2.21 and 3.42 µmol·h^−1^, respectively.^[^ ^21^ ^]^ In addition, piezoelectric‐catalytic Bi_4_Ti_3_O_12_ illustrates a high performance of ultrasonic‐supported organic pollutants degradation (MO, tetracycline hydrochloride, and bisphenol A) due to exhibiting production rates of 6.4 and 2.4 µmol·g^−1^·h^−1^ of powerful ^•^O_2_^−^ and ^•^OH radicals, respectively.^[^ ^63^ ^]^

### 3.4 Bismuth‐Based Materials

In recent years, significant advances have been achieved in improving photochemical performance trough polarization fields to increase charge transfer and reduce recombination. This has generated considerable interest in polarization‐enhanced photocatalysis, especially involving bismuth (Bi)‐based non‐centrosymmetric (NCS) materials. These compounds are known for their diversified compositions, distinctive electronic properties, and hybrid band structures. Many researchers have extensively reviewed Bi‐containing NCS materials related to polarization‐assisted photocatalysis, including their grouping, synthesis, and the mechanisms of carrier transfer associated with ferro‐, piezo‐, and pyroelectric effects.^[^ ^64^ ^]^ Recent progress in optimizing photocatalytic performance, along with current obstacles and future chances, has led to the use of bismuth‐based materials in various applications, such as photocatalytic N_2_O decomposition,^[^ ^65^ ^]^ organic pollutants photodegradation,^[^ ^66^ ^]^ removal of inorganic and organic pollutants,^[^ ^67^ ^]^ remediation of contaminated sites,^[^ ^68^ ^]^ and more. Bismuth‐based research has become a very popular field recently, offering exciting opportunities for new fundamental discoveries and applications, from innovative catalysts to new medicines (e.g., full‐spectrum‐driven photocatalysis,^[^ ^69^ ^]^ cancer,^[^ ^70^ ^]^ X‑ray detection,^[^ ^71^ ^]^ etc.), due to this heavy metal's nearly nonradioactive and non‐toxic properties.^[^ ^72^ ^]^ Furthermore, bismuth‐based materials have demonstrated advancements in supercapacitor technology,^[^ ^73^ ^]^ catalytic NH_3_ synthesis,^[^ ^74^ ^]^ environmental engineering solutions,^[^ ^75^ ^]^ and other areas.

### 3.5 Composite/Heterostructure Materials

Composites heterojunctions, known as piezo‐composites, have been accepted as a favorable material due to their outstanding and tunable performance. Piezo‐composite materials are especially suitable for underwater sonar and ultrasound transducers used in environmental applications.^[^ ^76^ ^]^ For example, In_2_Se_3_@Ag_3_PO_4_ achieved the “three birds with one stone”, including efficient degradation of 98.3% of ionic liquids, a concurrent H_2_ production yield of 582.7 µmol·g^−1^·h^−1^, and an elimination efficiency of 97.2% of U(VI).^[^ ^77^ ^]^ In addition, CdS/CdCO_3_‐CoS_2_ showed a notable H_2_ evolution and CO_2_ reduction rate of 64 867.88 and 654.7 µmol·g^−1^·h^−1^, respectively.^[^ ^78^ ^]^ In addition, SrBi_8_Ti_7_O_27_ nanosheets achieved a superior piezo‐photocatalytic efficiency of 361.34 µmol·g^−1^·h^−1^ of CO_2_‐to‐CO conversion and degradation performance with up to ∼100% of organic pollutants (100 mg L^−1^) within 60 minutes.^[^ ^79^ ^]^ Besides, 5% NaNbO_3_/WO_3_ reaches the decomposition efficiency of 73.7% of Rhodamine B within 120 minutes under the piezo‐photocatalysis condition.^[^ ^80^ ^]^ Interestingly, a hybrid catalyst of 0.7 BiFeO_3_‐0.3 BaTiO_3_ achieves a Rhodamine B degradation with a rate of 99% within 60 minutes under the combinatory effect of light irradiation and low‐frequency vibration.^[^ ^81^ ^]^ A novel catalytic SWCNT/ZnO/Fe_3_O_4_ composite has obtained impressive results, with a 94.19% cefixime degradation.^[^ ^82^ ^]^

As shown in Table 2, due to its large specific surface area, excellent electronic properties, and low cost, graphene‐based catalysts are emerging as potential photocatalysts. Nevertheless, the piezo‐photocatalytic H_2_ evolution of the composite is significantly limited by the rapid recombination of the photoinduced carriers. To optimize its performance, approaches such as heteroatom doping, microstructure engineering, defect creation, co‐catalyst decoration, and heterojunction construction are being developed. In particular, heterojunction‐based composites for the piezo‐photocatalyst exhibit impressive results in recent advancements in H_2_ generation and environmental remediation.

## 4 Dual‐Function Design Strategies

In the topics of piezo‐photocatalysis, co‐inducing and side‐by‐side design strategies rely on structural alterations, such as doping and defect engineering, to boost both piezoelectric and photocatalytic performance.^[^ ^12^ ^]^ These strategies also involve controlling morphology as SrTiO_3_/BaTiO_3_ nanofiber,^[^ ^104^ ^]^ using 2D/1D g‐C_3_N_5_/Bi_8_(CrO_4_)O_11_ nanostructures^[^ ^29^ ^]^ to enhance reactant contact and energy capture, and developing heterostructures^[^ ^80^ ^]^ to promote charge separation through the integration of Bi_4_Ti_3_O_12_@Carbon Schottky heterojunction,^[^ ^23^ ^]^ Z‐scheme Bi_2_WO_6_/Black TiO_2_ systems,^[^ ^96^ ^]^ or Bi_2_MoO_6_‐SOVs/MgFe_2_O_4_ S‐scheme.^[^ ^107^ ^]^ Combining these approaches links stress‐induced charge generation with light‐induced carrier generation, thereby increasing overall catalytic activity by enhancing carrier separation, transport, and access to reaction sites.^[^ ^10^ ^]^ Also, creating dual‐defect lattice sites in the crystal is a well‐known method of doping and defect engineering, involving oxygen vacancies or elemental dopants. This can disrupt the crystal symmetry and enhance piezoelectric behavior and carrier separation. For example, oxygen vacancy‐rich Bi_2_MoO_6_‐SOVs/MgFe_2_O_4_ demonstrated a decomposition efficiency of 96.8% for norfloxacin within 30 min.^[^ ^107^ ^]^ Additionally, structural modification can enhance the piezoelectric phenomena by creating an intrinsic electric field within the material, often through methods like polarization treatment. For instance, 2D CuInP_2_S_6_ crystals have been engineered to exhibit spin polarization and ferroelectric polarization, which improves photocatalytic CO_2_ reduction.^[^ ^28^ ^]^ Moreover, constructing piezo‐photocatalytic heterostructures is an effective way to create highly polar, porous structures and boost the piezoelectric response.^[^ ^7^ ^]^ Specifically, various strategies for generating synergistic effects have been investigated, including combining mechanical and optical activation, improving carrier separation and transport, and developing multi‐functional platforms.^[^ ^78^ ^]^ For example, In_2_Se_3_@Ag_3_PO_4_ has been implementing “Three‐in‐One” strategies,^[^ ^77^ ^]^ which involve designing materials that offer multiple synergistic effects, such as dual S‐scheme heterojunctions and interface electric fields, to significantly improve catalytic performance and achieve H_2_ production.

## 5 Piezo‐Photocatalysis for Hydrogen Evolution

In recent years, there has been significant development of new catalysts aimed at providing stable, highly efficient, and cost‐effective alternatives for H_2_ evolution and environmental remediation. For example, carbon‐modified electrochemical surfaces have been used for H_2_ production,^[^ ^108^ ^]^ carbon‐decorated NiMn_2_O_4_ has been utilized for azo dye degradation,^[^ ^109^ ^]^ Fe_3_O_4_/g‑C_3_N_4_/TiO_2_ catalyst has been employed for tetracycline degradation,^[^ ^110^ ^]^ and novel PANI/PVA‐NiCu has been supported for organic dyes removal.^[^ ^111^ ^]^ Besides, the authors have investigated efficient electrocatalysts for H_2_O_2_ generation,^[^ ^112^ ^]^ H_2_ adsorption,^[^ ^113^ ^]^ and pesticide decontamination.^[^ ^114^ ^]^

Interestingly, piezoelectric catalytic systems leverage the generated piezoelectric potential to modulate the energy bands of heterostructures, forming a piezoelectric field that promotes intermolecular electron and hole transfer and separation efficiency. Moreover, by decreasing the potential barrier height at their junction, the piezoelectric effect increases the population of electrons and holes. One of the most essential applications of piezoelectric catalysis is its ability to suppress the recombination of photo‐generated electrons and holes, thus improving the efficiency of photocatalytic processes. To overcome this challenge, the authors propose a novel complementary use of piezo‐coupled photocatalytic procedures to degrade pollutants, simultaneously recovering H_2_ through catalytic processes.

**Figure 3:**
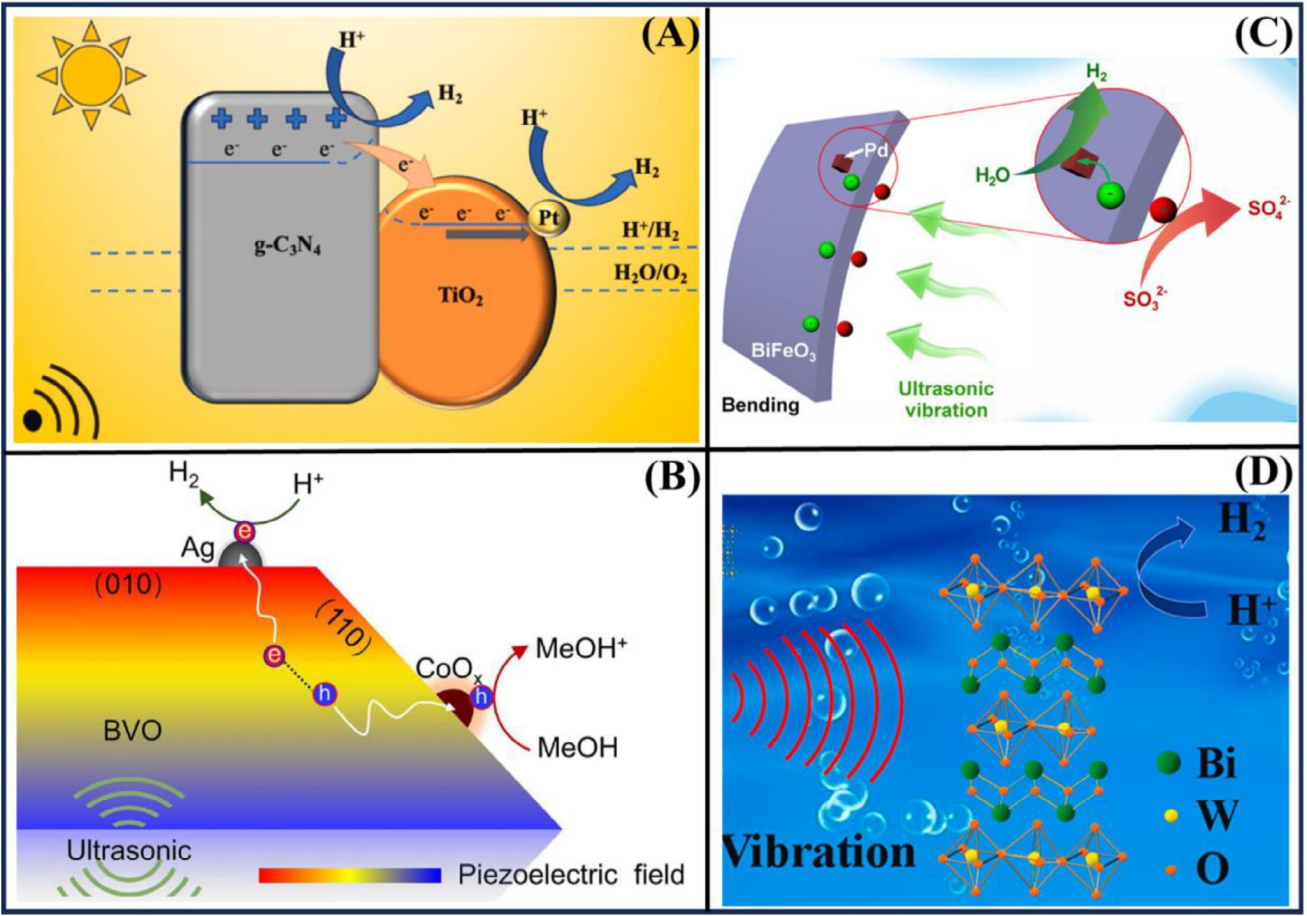
Various composites, such as (A) Pt/TiO_2_/g‐C_3_N_4_,^[^ ^94^ ^]^ (B) Ag‐BiVO_4_,^[^ ^115^ ^]^ (C) BiFeO_3_/Pd,^[^ ^16^ ^]^ (D) Bi_2_WO_6_,^[^ ^20^ ^]^ were employed for piezo‐photocatalytic H_2_ generation. Reproduced with permission.^[^ ^94^ ^]^ Copyright 2024, Elsevier. Reproduced with permission.^[^ ^115^ ^]^ Copyright 2023, Elsevier. Reproduced with permission.^[^ ^16^ ^]^ Copyright 2021, American Chemical Society. Reproduced with permission.^[^ ^20^ ^]^ Copyright 2020, Elsevier.

**Figure** **3**A shows a potential Pt/TiO_2_/g‐C_3_N_4_ material, suggesting a catalyst in sustainable H_2_ production. The authors have synthesized by an efficient one‐step calcination approach for obtaining a TiO_2_/g‐C_3_N_4_ composite. Well‐dispersed TiO_2_ nanoparticles and ultrathin flat g‐C_3_N_4_ nanosheets were prepared via a urea‐melamine mixture with a sol–gel method and subsequent annealing. The TiO_2_/g‐C_3_N_4_ as a catalyst exhibited a good performance, with lactic acid as a sacrificial reagent, the H_2_ generation rate was up to 32.5 mmol·g^−1^·h^−1^ when the 3 wt% Pt was supported on the TiO_2_/g‐C_3_N_4_. This increase was attributed to the efficient dispersion of TiO_2_ nanoparticles on g‐C_3_N_4_ and the built‐in heterojunction, which could promote the separation of charges. Particularly, ultrasonic treatment at the reaction liquid level further benefits from the piezoelectricity of g‐C_3_N_4_ nanosheets, and the H_2_ evolution rate of 43.57 and 2.9 mmol·g^−1^·h^−1^ were achieved under artificial sunlight and visible irradiation.^[^ ^94^ ^]^ Figure 3B demonstrates that facet and cocatalyst engineering were employed to enhance the efficiency of BiVO_4_. By tuning the pH value during the hydrothermal process, monoclinic BiVO_4_ catalysts with different exposed facets are produced. The resulting BiVO_4_ material, which has a high exposure of {110} facets, exhibits impressive H_2_ production activity of 617.9 µmol·g^−1^·h^−1^, surpassing most other BiVO_4_ materials due to its superior piezoelectric properties and strong charge transfer ability. The efficiency can be further increased by 44.7%, which facilitates effective charge alienation across the Ag‐BiVO_4_ surface. Moreover, it is shown that utilizing a CoO_x_ cocatalyst and methanol as a hole scavenger on the {110} surface doubles the H_2_ evolution efficiency, owing to improved charge separation and reduced water oxidation. This method is highly efficient, offering a novel approach to preparing advanced piezo‐catalysts.^[^ ^115^ ^]^

Figure 3C illustrates that the incorporation of cocatalysts, such as Pd, can greatly improve the piezo‐catalyst activity. Upon loading Pd on the BiFeO_3_ nanosheets, a prominent H_2_ evolution rate of 11.4 µmol·h^−1^ for 10 mg of catalyst was attained, which is 19 times higher than that of the bare BiFeO_3_. These activity correlations revealed that band tilting, exposed crystal plane, domain area, and loaded Pd amounts are the key parameters that directly impact activity.^[^ ^16^ ^]^ Figure 3D indicates that piezoelectric materials transform vibrations into electrical energy. The authors developed a novel piezo‐catalytic H_2_ generation process featuring with layer‐perovskite bismuth tungstate (Bi_2_WO_6_) nanoplates. The vibration energy harvesting led to a H_2_ productivity of 1147.8 µmol·g^−1^ of biomass in 6 h (191.3 µmol·g^−1^ of biomass·h^−1^). The spontaneous conduction band level of H^+^/H_2_ enables expedient water reduction to H_2_. This achievement realizes the optimally efficient use of the wasted energy and resolves energy shortage, which will draw more attention to the potential clean H_2_ energy for prospects ahead.^[^ ^20^ ^]^

**Figure 4:**
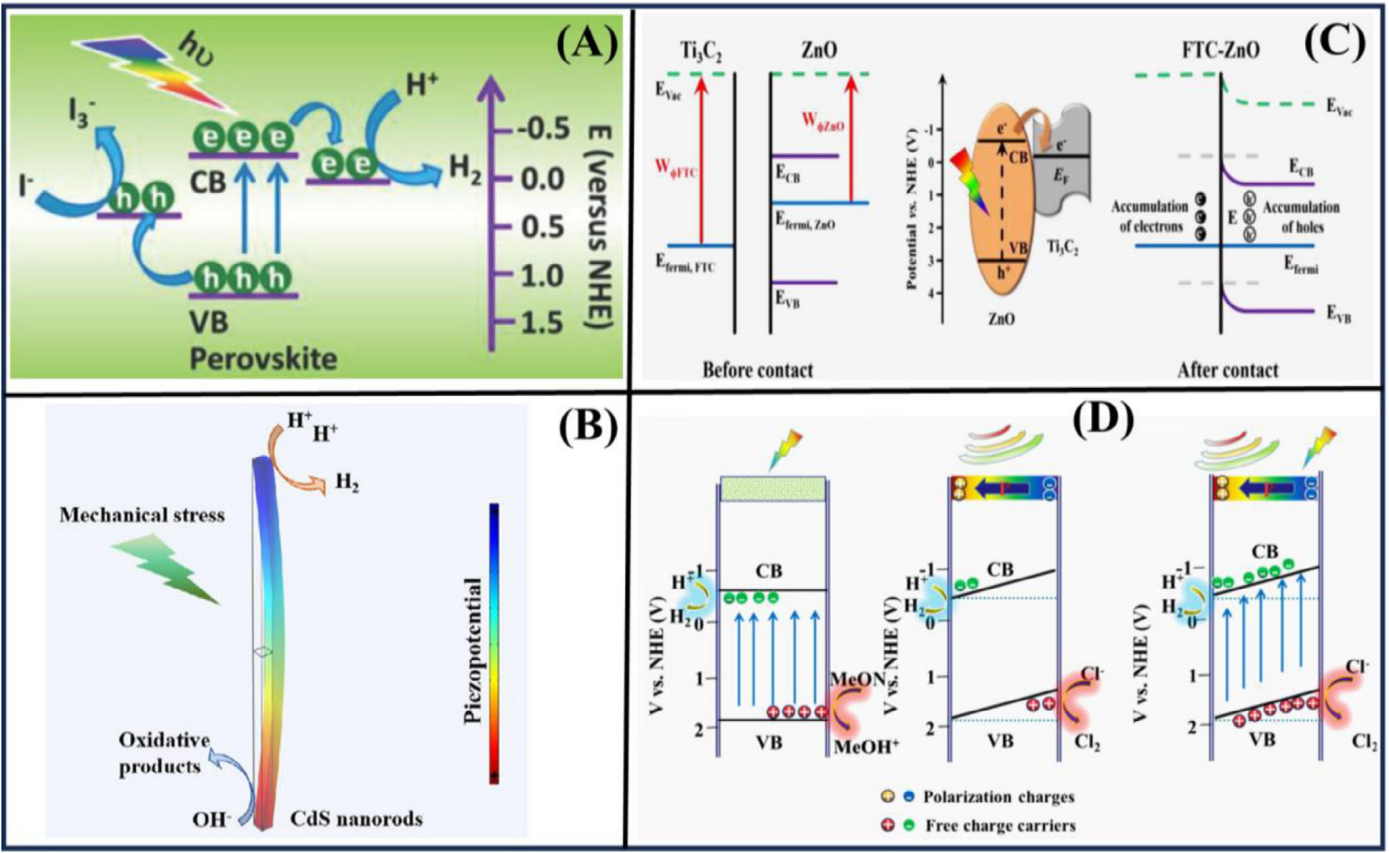
Remarkably boosted H_2_ evolution of (A) CH_3_NH_3_PbI_3_,^[^ ^21^ ^]^ (B) CdS,^[^ ^116^ ^]^ (C) Ti_3_C_2_T_x_‐ZnO,^[^ ^56^ ^]^ (D) La_2_NiO_4_.^[^ ^22^ ^]^ Reproduced with permission.^[^ ^21^ ^]^ Copyright 2019, John Wiley and Sons. Reproduced with permission.^[^ ^116^ ^]^ Copyright 2022, Elsevier. Reproduced with permission.^[^ ^56^ ^]^ Copyright 2022, Elsevier. Reproduced with permission.^[^ ^22^ ^]^ Copyright 2023, Elsevier.

**Figure** **4**A shows that the impressive coupling between the photo‐excitation of CH_3_NH_3_PbI_3_ and its piezoelectric effect applies to H_2_ production, with simultaneous ultrasonication and visible light irradiation. The CB minimum of CH_3_NH_3_PbI_3_ is even higher than the H_2_ evolution potential (0.046 V vs normal hydrogen electrode), guaranteeing highly efficient H_2_ evolution as well. It possesses a non‐centrosymmetric crystal structure and shows excellent piezoelectric properties, which can generate a built‐in electric field for facilitating the charge carrier separation. The experimental results reveal that the CH_3_NH_3_PbI_3_ powders exhibit a high piezo‐photocatalytic H_2_ generation rate of 23.30 µmol h^−1^ in a hydroiodic acid solution under simultaneous light and mechanical actions. This value is much larger than the rate of piezo‐catalytic (2.21 µmol h^−1^) and photocatalytic (3.42 µmol h^−1^), as well as their total sum (5.63 µmol h^−1^). This novel concept of recombination control of charge carriers has made a new world record of photocatalytic activity.^[^ ^21^ ^]^ CdS nanorods, with ultrasonic vibration, showed a remarkable piezo‐catalytic H_2_ production rate of 157 µmol·g^−1^·h^−1^, ≈ 2.8 times as high as that of the CdS nanospheres, as shown in Figure 4B. This increased activity was directly associated with the high piezoelectric coefficient of the nanorods and better mechanical energy‐harvesting, leading to effective alienation of the charge carriers. The results distinctly demonstrate a new route to the creation of superior piezo‐catalytic materials via the rational control of microstructures.^[^ ^116^ ^]^

Figure 4C reports a new type of 2D interfacial Schottky heterojunction formed by the introduction of ultrathin Ti_3_C_2_T_x_ deposited on ZnO films, where piezo‐polarization charges induced by piezoelectricity are utilized to separate photogenerated electron‐hole pairs. These charges are also responsible for the interface redox reactions in the piezo‐photocatalysis process. In addition, the dissipation of light refracted at the metal‐semiconductor surface is lessened, resulting from the piezoelectric polarization of the incorporated electric field. The combination of Ti_3_C_2_T_x_ and ZnO, together with a 2D‐layered MXene layer and Schottky electric field, enhances photocatalytic H_2_ evolution upon illumination and ultrasonic irradiation. These results may further widen the application of ZnO films as piezo‐semiconductors in H_2_ evolution and provide suggestions for developing new piezo‐photocatalysts with metallic MXenes.^[^ ^56^ ^]^ As shown in Figure 4D, 2D perovskite oxides, especially acidified La_2_NiO_4_, exhibit strong ferroelectricity and piezoelectricity, endowing them with high light responsiveness and excellent chemical stability. Acidified La_2_NiO_4_ under full‐spectrum ultrasonic/marine seawater irradiation reached an impressive rate of H_2_ production being 1315.44 µmol·g^−1^·h^−1^, which is over 200 times higher than that of pure La_2_NiO_4_ in CH_3_OH. The noticeable enhancement in efficiency is attributed to enhanced charge carrier alienation. In addition, inorganic salts contained in seawater may promote H_2_ generation as sacrificial agents. This work not only expands knowledge of piezo‐photocatalysis, but also demonstrates a new benchmark for achieving optimized influence of multifield coupling on H_2_ evolution.^[^ ^22^ ^]^

**Figure 5:**
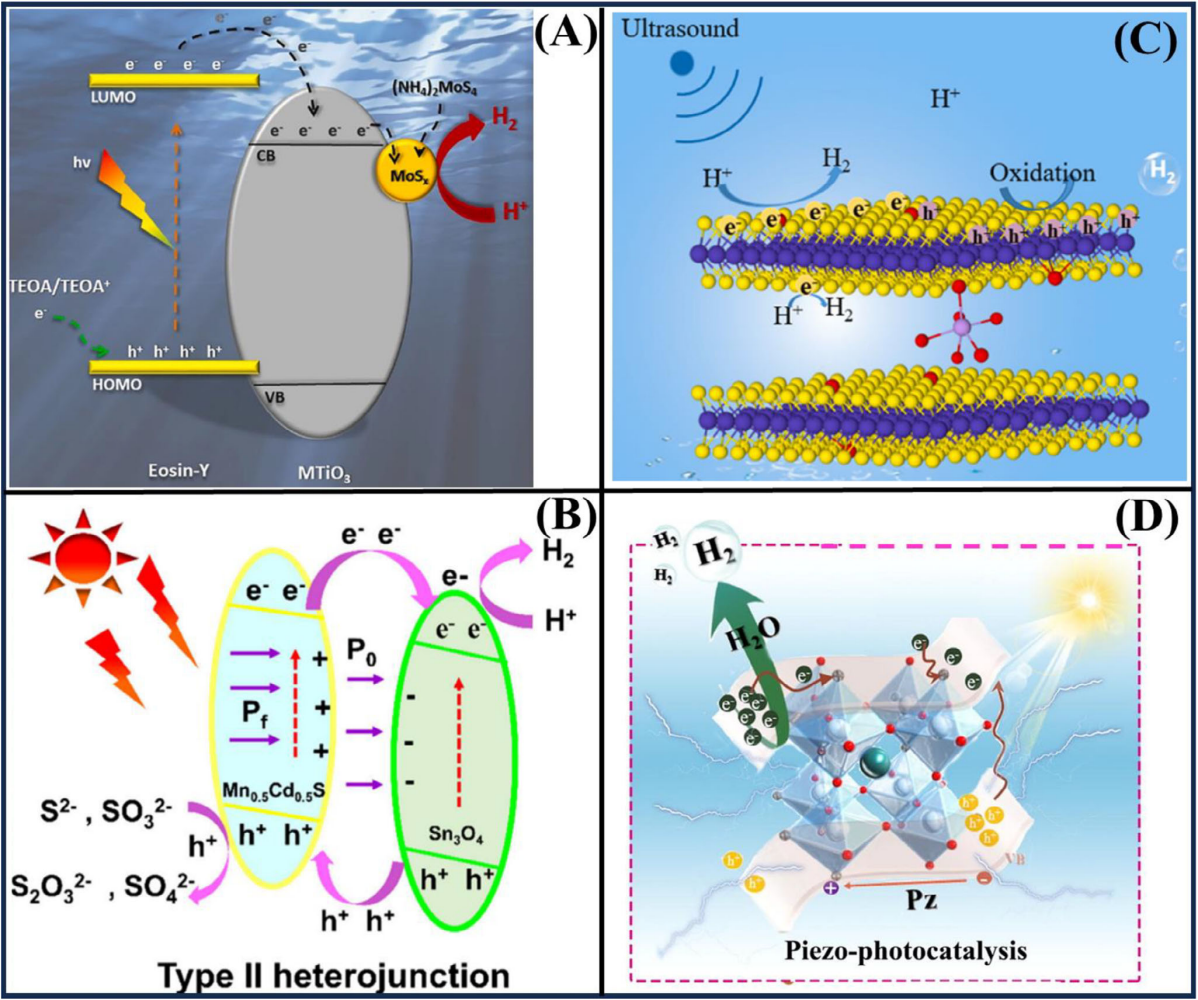
H_2_ evolution using (A) photocatalytic MTiO_3_ (M: Ba, Sr)/MoS_x_,^[^ ^93^ ^]^ (B) piezo‐photocatalytic Sn_3_O_4_/Mn_0.5_Cd_0.5_S,^[^ ^32^ ^]^ (C) O‐doped MoS_2_,^[^ ^19^ ^]^ (D) OH‐modified SrTiO_3_.^[^ ^6^ ^]^ Reproduced with permission.^[^ ^93^ ^]^ Copyright 2023, Elsevier. Reproduced with permission.^[^ ^32^ ^]^ Copyright 2024, American Chemical Society. Reproduced with permission.^[^ ^19^ ^]^ Copyright 2022, Elsevier. Reproduced with permission.^[^ ^6^ ^]^ Copyright 2022, Elsevier.

**Figure** **5**A demonstrates that perovskite‐related materials MoS_x_, deposited on the MTiO_3_ (M: Ba, Sr), have emerged as one of the most promising materials for H_2_ evolution in solar light. The H_2_ evolution efficiencies and stabilities of the BaTiO_3_/MoS_x_ and SrTiO_3_/MoS_x_ are both largely superior to the pristine MTiO_3_, and can be positively ordered according to the performances: SrTiO_3_/MoS_x_ > BaTiO_3_/MoS_x_ > MoS_x_ > SrTiO_3_ > BaTiO_3_. Furthermore, under the same reaction conditions, SrTiO_3_/MoS_x_ all the time performs better H_2_ activity than that of SrTiO_3_/Pt, due to a larger amount of active sites that originate from MoS_x_ introduced on the surface of the catalyst by Mo–S and S–S bonds.^[^ ^93^ ^]^ Figure 5B illustrates that piezo‐photocatalytic H_2_ production via Sn_3_O_4_/Mn_0.5_Cd_0.5_S reached a noteworthy rate of 21.46 mmol·g^−1^·h^−1^, after the process of ultrasonic treatment and irradiation, which was about 3.85 times higher than that of pristine Mn_0.5_Cd_0.5_S. These catalysts also showed excellent stability with a sustained rate of 19.39 mmol·g^−1^·h^−1^ after four cycles. The piezo‐photocatalytic process and the establishment of the heterojunction resulted in an electric field, and this proved beneficial in photo‐derived charge separation and transport, thus driving more H_2_ evolution. This work may offer a successful scheme for the rational design of type‐II heterojunction catalysts that can harvest both mechanical and solar light energy to produce sustainable H_2_.^[^ ^32^ ^]^

Figure 5C significantly shows MoS_2_ through oxygen doping, largely suppressing the layer‐number‐dependent piezoelectric response and driving the system to an out‐of‐plane polarization. As a consequence, O‐doped MoS_2_ has increased piezoelectricity at all numbers of layers. Higher charge carrier concentration, enhanced Hall mobility, and lower resistivity due to the enhancements in piezoelectric response. The O‐doped MoS_2_ exhibits an excellent H_2_ production rate of 47.75 µmol·g^−1^·h^−1^ under pure‐water conditions, which is higher than that of MoS_2_ (20.19 µmol·g^−1^·h^−1^) without any modification. This research offers a new scheme for modulating the piezoelectric performance of transition metal dichalcogenides and thus also provides a new possibility for the design of high‐efficiency piezo‐catalysts.^[^ ^19^ ^]^ Figure 5D introduces that OH‐modified SrTiO_3_ is employed for catalytic applications in piezo‐photocatalytic H_2_ production. The super hydrophilic property of OH‐modified SrTiO_3_ can remarkably increase the interface between water molecules and TiO_2_ particles, and the enlarged oxygen vacancy functions as a barrier for effective electron‐hole separation. The H_2_ evolution efficiencies of all the reactions with OH‐functionalized SrTiO_3_ are thus almost two times higher than those of pristine SrTiO_3_. Among them, the maximum piezo‐photocatalytic H_2_ production rate for OH‐modified SrTiO_3_ as a function of pressure reaches an outstandingly high value of 701.2 µmol·g^−1^·h^−1^, which is 5.3 times greater than that of the photocatalytic performance of SrTiO_3_. This study shows an efficient method of fabricating the functional group modified materials with superior piezo‐photocatalytic activity.^[^ ^6^ ^]^

The synergistic effect of piezoelectric and photocatalytic activity in these bimodal systems leads to performance that surpasses that of each process alone. The piezoelectric effect further improves not only significantly higher photodegradation rates for various pollutants but also supports essential reactions, particularly hydrogen production, making these systems potentially revolutionary for addressing energy and environmental challenges. By integrating the strengths of piezo‐catalysis and photocatalysis, these systems open new opportunities for efficient, eco‐friendly treatments across many fields.

## 6 Piezo‐Photocatalytic CO_2_ Reduction

**Figure 6:**
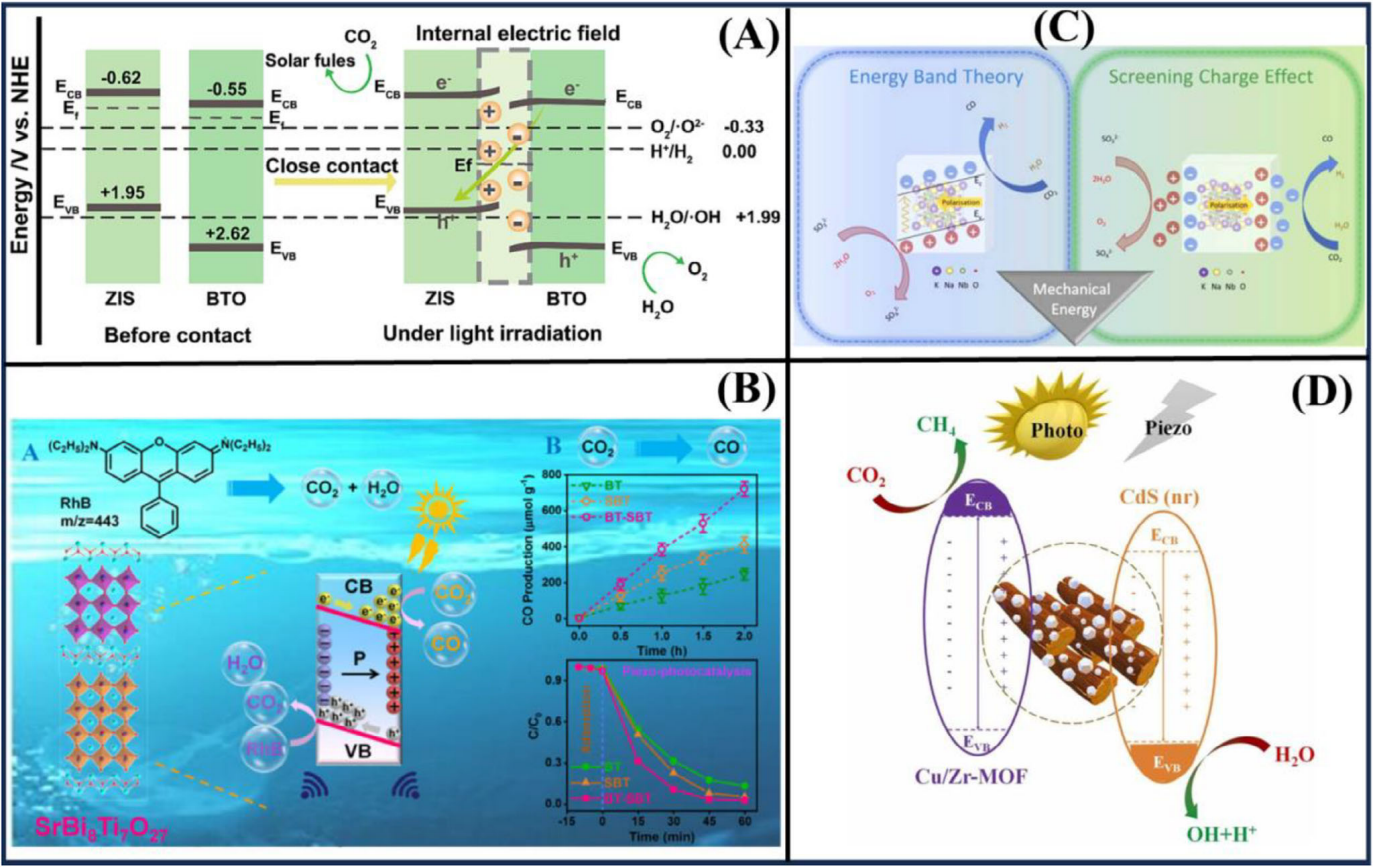
Various mechanisms of the CO_2_ reduction employing (A) ZnIn_2_S_4_/BaTiO_3_,^[^ ^117^ ^]^ (B) SrBi_8_Ti_7_O_27_,^[^ ^79^ ^]^ (C) (K_0.5_Na_0.5_)_0.97_Li_0.03_NbO_3_,^[^ ^118^ ^]^ (D) Cu/Zr‐MOF@CdS.^[^ ^119^ ^]^ Reproduced with permission.^[^ ^117^ ^]^ Copyright 2024, Elsevier. Reproduced with permission.^[^ ^79^ ^]^ Copyright 2024, American Chemical Society. Reproduced with permission.^[^ ^118^ ^]^ Copyright 2022, Elsevier. Reproduced with permission.^[^ ^119^ ^]^ Copyright 2024, Elsevier.

**Figure** **6**A indicates that ZnIn_2_S_4_/BaTiO_3_ direct Z‐scheme heterojunction successfully reduces CO_2_ to CO with a high yield of 105.89 µmol·g^−1^·h^−1^, which was about 2.55 and 3.62 times that of ZnIn_2_S_4_ and BaTiO_3_, respectively. This study emphatically proves that the excellent photo‐catalytic performance derives from the combinatory effect between the piezoelectric and Z‐scheme electron transfer that boosts the charge‐separation and electron‐radiation. This next‐generation approach is expected to transform strategies for CO_2_ reduction and facilitate progress in sustainable energy production.^[^ ^117^ ^]^ As shown in Figure 6B, the doped ferroelectric‐like SrBi_8_Ti_7_O_27_ intergrowth nanosheets present a highly efficient and benign piezo‐photocatalyst for CO_2_ reduction reaction and contamitant degradation. The performance of this material is much better than that of single ferroelectric materials, with a CO_2_‐to‐CO conversion rate of 361.34 µmol·g^−1^·h^−1^. Further, it can efficiently decompose high concentrations (100 mg.L^−1^) of organic pollutants (≈100% in 60 min), which is much higher than most of the formerly reported piezo‐photocatalysts. These results suggest the promising application prospects of intergrowth ferroelectric materials in sustainable energy and environmental science.^[^ ^79^ ^]^

Figure 6C shows novel discoveries of the piezo‐catalytic CO_2_ reduction using (K_0.5_Na_0.5_)_0.97_Li_0.03_NbO_3_. By employing ultrasound to produce pressure waves in a suspension of these ceramic particles, we greatly promote the spontaneous polarization of the (K_0.5_Na_0.5_)_0.97_Li_0.03_NbO_3_, producing surface charges that promote efficient CO_2_ reduction. We systematically study the effects of CO_2_ concentration, dissolved species, and catalyst loading on the performance in this work. By careful optimization, the authors were able to reach an outstanding CO_2_ reduction rate of 438 µmol·g^−1^·h^−1^, much higher than rates previously reported via pyro‐catalytic approaches. This powerful and controllable piezo‐catalytic tactic is perfectly ready to revolutionize CO_2_ reduction by applying the vibrational energy, which will manifest considerable environmental contributions.^[^ ^118^ ^]^ Figure 6D introduces CO_2_ methanation via Cu/Zr‐MOF@CdS as a method to enhance selectivity for CH_4_ and improve CO_2_ conversion by optimizing the electron transfer process. Copper doping in Zr‐MOF suppresses carbon‐carbon coupling and facilitates *CO protonation to *HCO. Furthermore, the piezoelectric properties of both CdS and Zr‐MOF induce a type (II) to Z‐scheme transition, which aids in charge separation. The CH_4_ evolution rate at 80% CO_2_ conversion in the photocatalytic process was 23.6 µmol·g^−1^·h^−1^. After the piezo‐photocatalytic process was initiated, these figures increased to 52.2 µmol·g^−1^·h^−1^ and 99% CO_2_ conversion. Effective reaction pathways for piezo‐photocatalytic CO_2_ methanation in this study are proposed through in situ measurements.^[^ ^119^ ^]^

**Figure 7:**
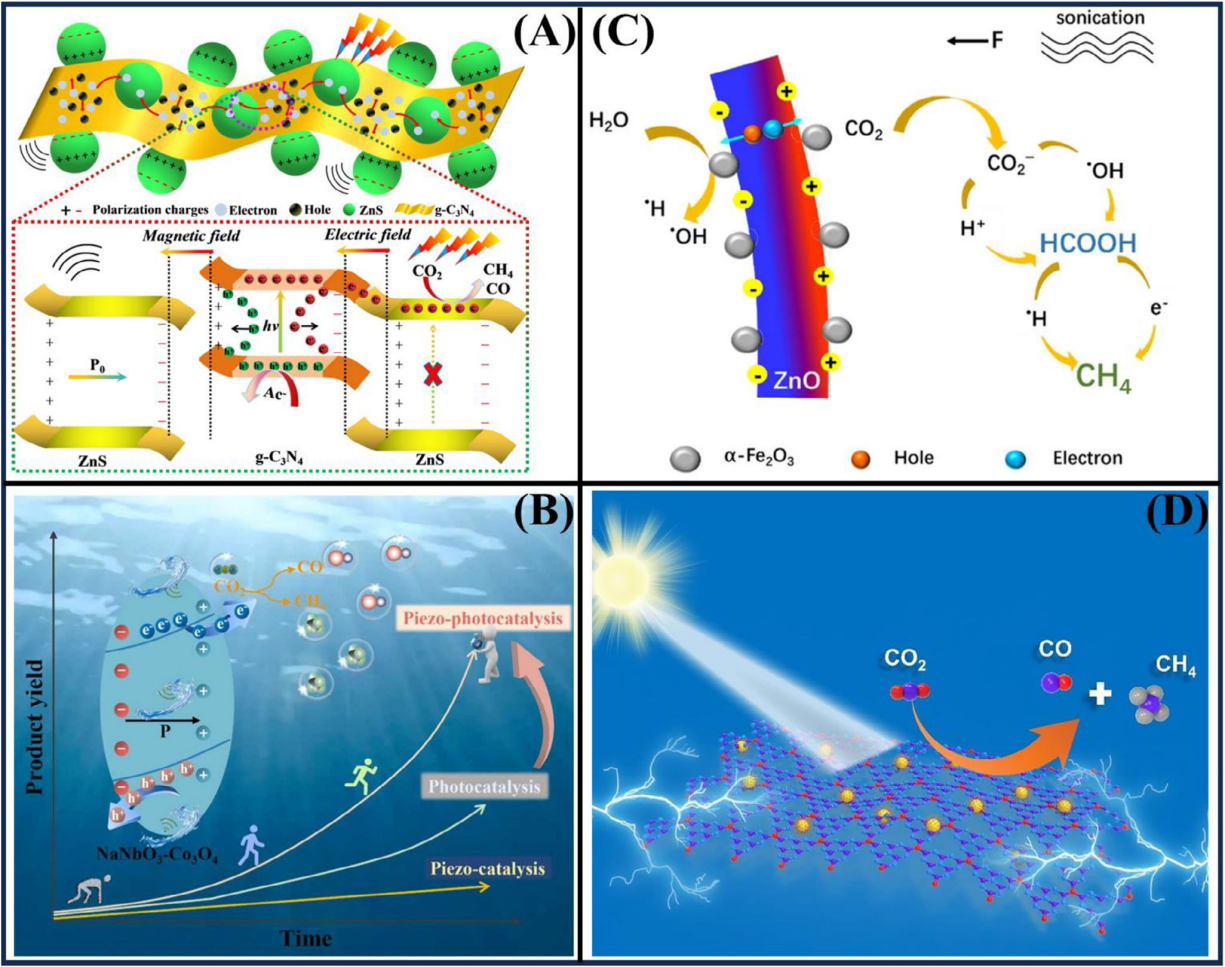
Piezo‐photocatalysis for CO_2_ reduction via (A) ZnS/g‐C_3_N_4_,^[^ ^120^ ^]^ (B) NaNbO_3_‐Co_3_O_4_,^[^ ^121^ ^]^ (C) α‐Fe_2_O_3_/ZnO,^[^ ^122^ ^]^ (D) Au_25_(p‐mercaptobenzoic acid)_18_ nanoclusters modified red g‐C_3_N_4_.^[^ ^57^ ^]^ Reproduced with permission.^[^ ^120^ ^]^ Copyright 2022, John Wiley and Sons. Reproduced with permission.^[^ ^121^ ^]^ Copyright 2024, Elsevier. Reproduced with permission.^[^ ^122^ ^]^ Copyright 2024, Springer Nature. Reproduced with permission.^[^ ^57^ ^]^ Copyright 2023, Elsevier.

To promote bulk and surface carrier separation in g‐C_3_N_4_ for CO_2_ reduction and achieve efficient CH_4_ production, **Figure** **7**A displays a g‐C_3_N_4_ modification with dual‐functional ZnS. ZnS acts as a co‐catalyst that captures photogenerated electrons from g‐C_3_N_4_ and generates polarization electric fields, facilitating the fast migration of charges to the surface. The ZnS/ g‐C_3_N_4_ nanocomposite showss high piezo‐photocatalytic activity for CO_2_ reduction under simultaneous vibration and visible‐light irradiation, outperforming the individual photocatalytic and piezo‐catalytic processes. With the optimal mass ratio of ZnS to g‐C_3_N_4_, the authors achieve an impressive selectivity of 95.7% for CO_2_ reduction to CH_4_ when the ZnS content is 7.95 wt%. This work sets a new record in regulating carrier pathways and enhancing charge separation in semiconductors for selective CO_2_ conversion.^[^ ^120^ ^]^ Figure 7B introduces a new approach, Co_3_O_4_ into NaNbO_3_ through the photo‐reduction can distort Nb‐O octahedra, leading to the improvement of piezoelectric property, and generate more Co‐active sites for the CO_2_ adsorption and perovskite to reduce the reaction energy barrier. As a consequence, the CO yield reaches ≈ 4579.71 µmol·g^−1^ in the presence of ultrasound and visible light for NaNbO_3_‐Co_3_O_4_ nanocubes.^[^ ^121^ ^]^

Figure 7C reports on the synthesis of a new heterojunction material based on the joints of the α‐Fe_2_O_3_ nanoparticles on ZnO microrod surfaces via a hydrothermal process. By tuning the α‐Fe_2_O_3_ amount on ZnO, the activity of CO_2_ reductions was increased from 8.5 to 118.2 µmol·g^−1^·h^−1^, for CH_4_ from 32.9 to 18.4 µmol·g^−1^·h^−1^, and for CHOOH, and CH_4_ selectivity was enhanced from 20.6% to 86.5%. These results suggest that the α‐Fe_2_O_3_ promotes charge separation and CO_2_ adsorption, and thus improves the reduction of CO_2_ to CH_4_. This study provides a tactful strategy to enhance CO_2_ reduction performance with product yield in piezoelectric catalysis.^[^ ^122^ ^]^ Figure 7D shows Au_25_(p‐mercaptobenzoic acid)_18_ nanoclusters modified red g‐C_3_N_4_ with in situ seed growth. The introduction of Au_25_(p‐mercaptobenzoic acid)_18_ nanoclusters can generate more active sites and a long‐distance electric field, to stretch out the photoexcited carriers during the photo‐piezoelectric excitation. Thus, the CO_2_ reduction rate to CO on Au_25_(p‐mercaptobenzoic acid)_18_ nanoclusters modified red g‐C_3_N_4_ could be up to 111.95 µmol·g^−1^·h^−1^ of CO, over 3 times enhanced than that of pure red g‐C_3_N_4_. This work demonstrates the power of metal cluster‐loaded semiconductors for the significantly increased piezo‐photocatalytic CO_2_ reduction.^[^ ^57^ ^]^

## 7 Piezo‐Photocatalytic Degradation of Organic Pollutants

According to the perspective of sustainable development, simultaneous hydrogen evolution production alongside environmental remediation or elimination of substance pollutants is a promising avenue for handling environmental issues and the energy crisis. In conventional photocatalytic H_2_ production processes, extensive researches have focused on the reduction reaction capability and mechanistic aspect of photo−generated electrons; however, less attention has been paid to the simultaneous oxidation processes involving holes. Historically, sacrificial reagents, whether toxic organic (e.g., methanol) or inorganic (e.g., sulfides), have been used to fill these holes. On the other hand, the need for supplementary sacrificial reagents increases the cost of H_2_ production and poses the risk of harmful substances, which is undesirable in practical applications. Organic pollutants found in wastewater may also possess additional value if recognized correctly. A very promising and attractive strategy is to replace sacrificial agents with chemical contaminants to enhance H_2_ production. Therefore, efficient degradation of contaminants with concurrent H_2_ recovery can meet the urgent need for constructing a photocatalytic system for wastewater treatment.

**Figure 8:**
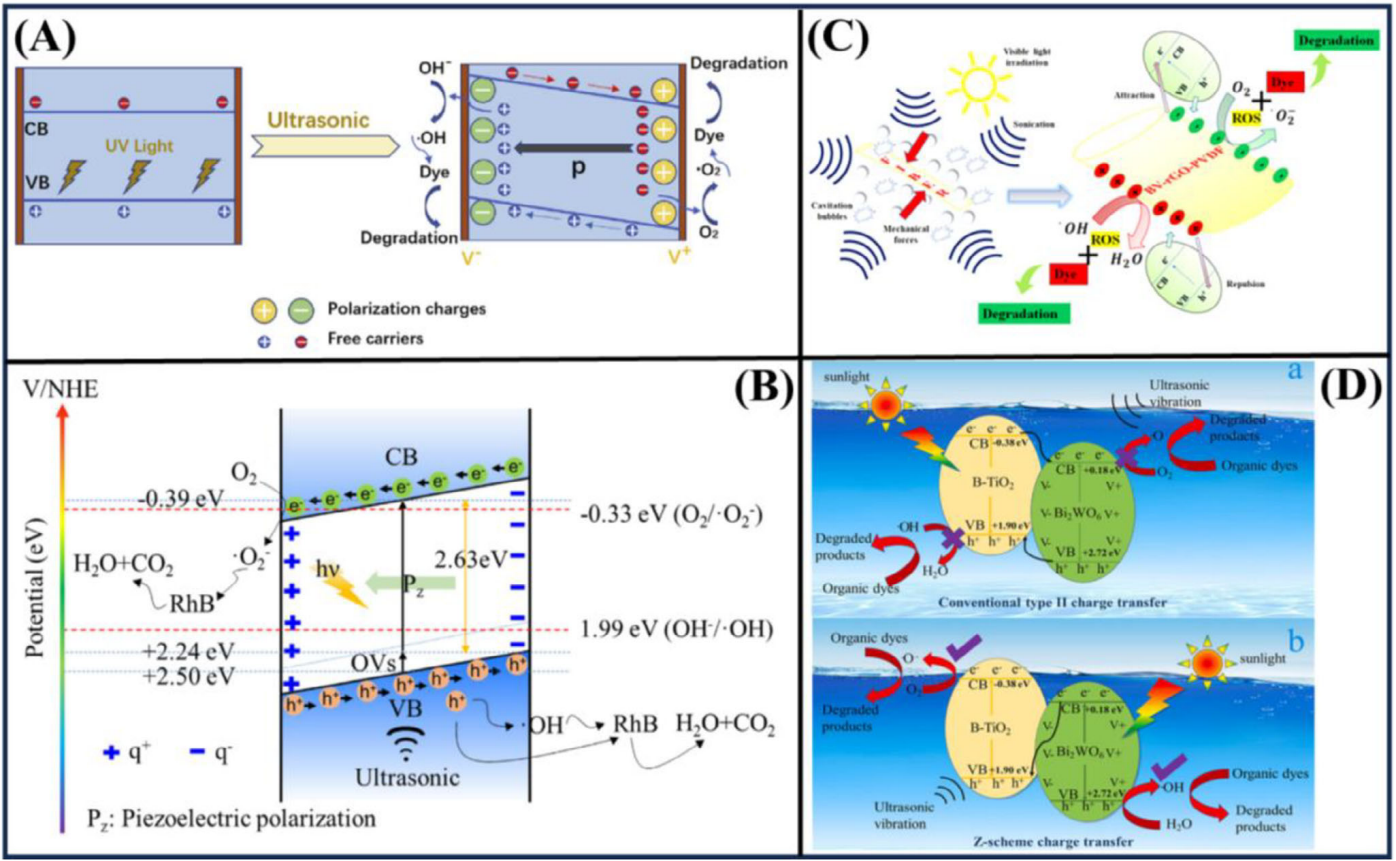
Various mechanisms of the organic degradation via (A) BaTiO_3_,^[^ ^15^ ^]^ (B) AgNbO_3_,^[^ ^86^ ^]^ (C) BiVO_4_‐reduced graphene oxide‐polyvinylidene fluoride,^[^ ^97^ ^]^ (D) Bi_2_WO_6_/TiO_2_.^[^ ^96^ ^]^ Reproduced with permission.^[^ ^15^ ^]^ Copyright 2020, Elsevier. Reproduced with permission.^[^ ^86^ ^]^ Copyright 2022, Elsevier. Reproduced with permission.^[^ ^97^ ^]^ Copyright 2023, Elsevier. Reproduced with permission.^[^ ^96^ ^]^ Copyright 2022, Elsevier.

**Figure** **8**A introduces BaTiO_3_ nanowires for the degradation of 5 mg·L^−1^ methyl orange (MO) in an aqueous solution (5 mL) through the integration of photocatalysis with the piezoelectric effect under UV irradiation and ultrasonic vibration. The authors reached an impressive destruction rate of ≈ 98.17% in as little as 80 min, which is much more efficient than piezo‐catalysis or photocatalysis alone. The radical trapping tests verified that hydroxyl (^•^OH) and superoxide (^•^O_2_^−^) radicals were present, thus indicating the significant contribution of these radicals to MO degradation. In addition, consecutive cycles only caused a minor (15%) loss in performance after four cycles. This research is the basis of the piezo‐photocatalytic materials, which could be applied toward environmental protection.^[^ ^15^ ^]^ Figure 8B shows that oxygen vacancies are introduced into AgNbO_3_ by annealing in nitrogen (AgNbO_3_‐N_2_). The as‐formed AgNbO_3_‐N_2_ featured enhanced piezo‐photocatalytic activity in Rhodamine B (RhB) degradation compared with AgNbO_3_ without vacancies. The authors find that AgNbO_3_‐N_2_ decreases the bandgap, improves UV–vis light adsorption ability, and accelerates the piezo‐photocatalytic performance. This work highlights the importance of oxygen vacancies for increased efficiency.^[^ ^86^ ^]^

As shown in Figure 8C, the authors utilized the electrospinning technique to fabricate three different types of membranes: polyvinylidene fluoride (PVDF), bismuth vanadate‐PVDF (BV‐PVDF), and BV‐reduced graphene oxide‐PVDF (BV‐rGO‐PVDF) composite. Unlike the expensive noble metals (e.g., platinum), rGO is a versatile, low‐cost, and environmentally friendly material for enhancing photocatalytic performance. The membranes were then tested as a catalyst in a 10 mL solution of 5 mg·L^−1^ methylene blue (MB) dye to assess the correlation between their structure and catalytic performance. Scavengers such as ethylenediaminetetraacetic acid (EDTA), benzoquinone (BQ), and isopropanol (IPA) were also employed to investigate degradation intermediates. The results indicate that BV‐rGO‐PVDF exhibits remarkable piezo‐catalytic and photocatalytic performance compared to both PVDF and BV‐PVDF. It achieved an impressive 82% dye degradation in under 180 min (degradation constant of 0.0088 min^−1^). The findings demonstrate the effectiveness of this novel hybrid material in degrading organic pollutants.^[^ ^97^ ^]^ Figure 8D presents a Bi_2_WO_6_/Black TiO_2_ (Bi_2_WO_6_/B–TiO_2_) heterojunction that behaves as a Z‐scheme system with remarkable piezo‐photocatalytic performance. In particular, the optimal Bi_2_WO_6_/B–TiO_2_ shows a significantly high degradation efficiency of 98.43% for the cationic dye RhB within 60 min, aided by both ultrasonication and sunlight. This efficiency is much higher than that of separate photodegradation (54.23%) and piezo‐degradation performance (26.33%). This unique hybrid is also capable of degrading anionic dyes such as methyl orange and the antibiotic diclofenac sodium. This work not only demonstrates an effective catalyst development strategy but also integrates the piezoelectric effect with photocatalysis in a novel fashion.^[^ ^96^ ^]^

One of the main difficulties of traditional photocatalysis is that the photoproduced electron‐hole pairs rejoin quickly, which seriously reduces overall efficiency. When exposed to light, a photocatalyst produces electron‐hole pairs that should ideally participate in redox reactions for degradation or conversion. However, the recombination of these charge carriers before they can act makes the photocatalytic reaction inefficient. To address this, some dual piezo‐photocatalytic systems use mechanical stress, such as mass, vibration, or ultrasound. This mechanical response generates a piezoelectric potential within the system, creating an internal electric field. This field plays a crucial role by helping to prevent the recombination of photogenerated charges, such as electrons and holes, by effectively separating them. As a result, more charge carriers are preserved to drive catalytic reactions, which enhances the electrocatalytic activity.

**Figure 9:**
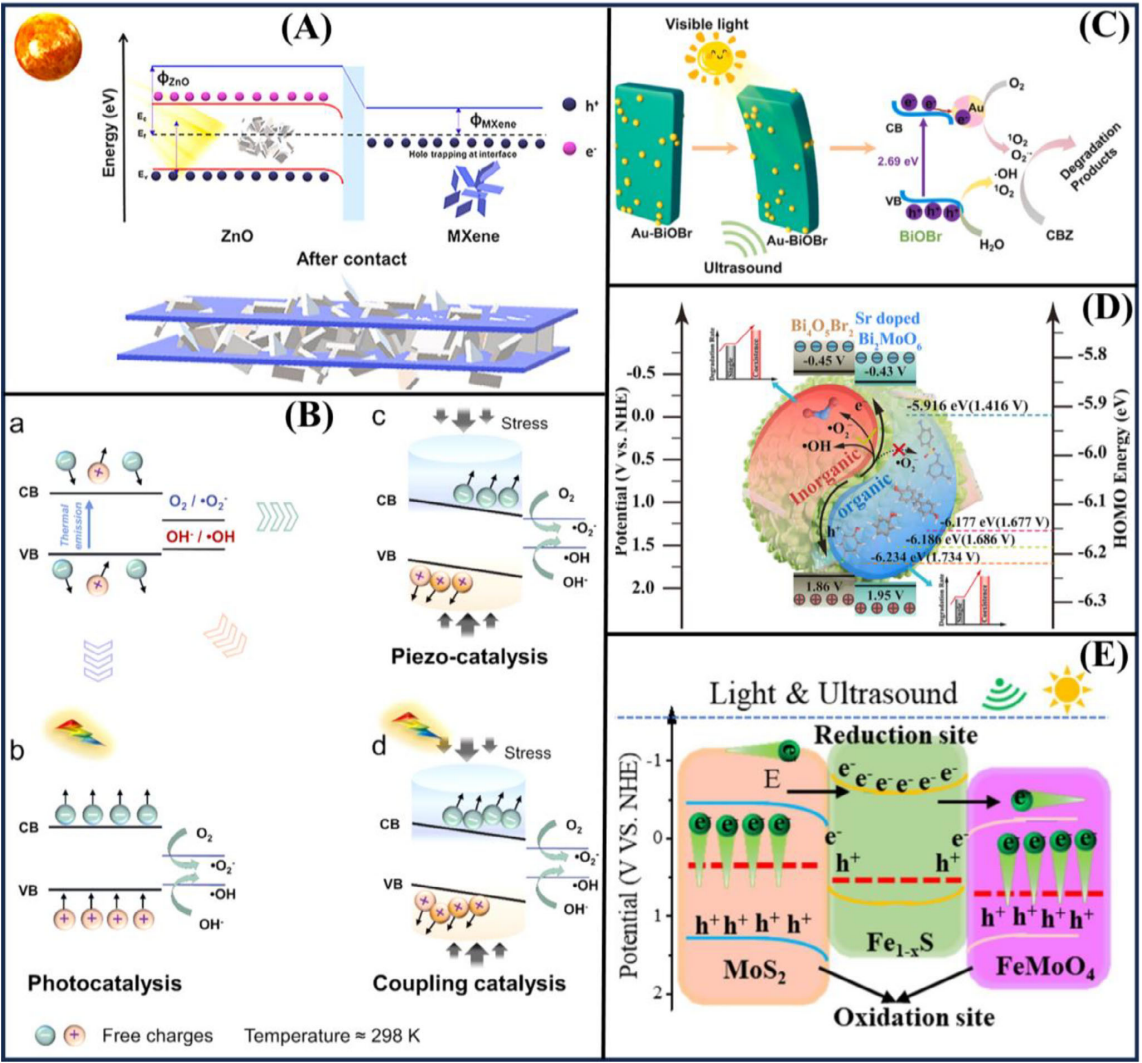
Degradation of various pollutant via photocatalysis of (A) ZnO/Ti_3_C_2_T_x_,^[^ ^90^ ^]^ (B) piezo‐photocatalysis of 0.7 BiFeO_3_‐0.3 BaTiO_3_,^[^ ^81^ ^]^ (C) Au‐BiOBr,^[^ ^102^ ^]^ (D) Sr‐doped Bi_4_O_5_Br_2_/Bi_2_MoO_6_,^[^ ^98^ ^]^ E) Fe_1−x_S/FeMoO_4_/MoS_2_.^[^ ^123^ ^]^ Reproduced with permission.^[^ ^90^ ^]^ Copyright 2021, Elsevier. Reproduced with permission.^[^ ^81^ ^]^ Copyright 2024, Elsevier. Reproduced with permission.^[^ ^102^ ^]^ Copyright 2022, Elsevier. Reproduced with permission.^[^ ^98^ ^]^ Copyright 2023, Elsevier. Reproduced with permission.^[^ ^123^ ^]^ Copyright 2025, Elsevier.

Nanotechnology presents an excellent opportunity to purify the increasingly prevalent contaminants in water. An important approach for degrading industrial dyes is photocatalysis using low‐cost photocatalysts. MXene is a potential photocatalyst but is mostly limited by the re‐stacking of its flakes and the electron‐hole recombination due to its low bandgap. As shown in **Figure** **9**A, to address these issues, the authors have fabricated 2D/2D‐composite nanoarchitectures by integrating zinc oxide nanoflakes with MXene (ZnO/Ti_3_C_2_T_x_). This composite can efficiently decompose 99.9% MB dye in 40 min and exhibits good antibacterial performance. These results will greatly benefit researchers in exploring hybrid 2D‐2D composite nanoarchitectures for several applications.^[^ ^90^ ^]^ Figure 9B shows that 0.7 BiFeO_3_‐0.3 BaTiO_3_ hybrid catalyst exhibits a remarkable 99% of RhB solution degradation at 60 min with simultaneously applied low‐frequency vibrations and light irradiation, and a piezoelectric performance enhancement of 148%. The internal electric field derived from the mechanical stress drives the band structure modulation. It adjusts the photoexcited carrier separation, as well as the generation of reactive oxygen species, leading to efficient pollutant degradation.^[^ ^81^ ^]^

The piezo‐photocatalytic activity of gold‐decorated bismuth oxybromide (Au‐BiOBr) has been comprehensively studied to shed light on charge carrier dynamics and to optimize the coupling of dual energy sources for environmental remediation. The piezoelectric effect, photo‐response, and charge separation efficiency were examined in Au‐BiOBr to explore piezoelectric effect‐assisted photocatalysis, while the carbamazepine (CBZ) degradation was used as a reflection of the catalytic performance, as shown in Figure 9C. The piezo‐photocatalytic degradation efficiency of CBZ was phenomenal, 95.8% in 30 min, with a rate constant being 1.73‐fold higher compared to piezo‐ and photo‐catalysis alone. These effects are the piezoelectric phenomenon of BiOBr that acts to enhance the incorporated electric field and to modify the band structure desirably, as well as the promotion of charge transfer and light absorption by gold nanoparticles. This work opens a promising approach for the solar‐mechanical‐driven environmental remediation.^[^ ^102^ ^]^ Figure 9D shows a Sr‐doped Bi_4_O_5_Br_2_/Bi_2_MoO_6_ heterojunction catalyst that exhibited an excellent removal efficiency for nitrite (91.7%) and 4‐chlorophenol (4‐CP) (100%) at 60 min, which were 3.56 times and 2.19 times of the pristine Bi_4_O_5_Br_2_ and Bi_2_MoO_6_, respectively. The results show that the increased catalytic activity is due to the piezoelectric effect, strontium phenomenon, and heterojunction structure, which facilitate the alienation of charges. There was also good stability of the decomposition activity of the Sr‐doped Bi_4_O_5_Br_2_/Bi_2_MoO_6_/polyacrylonitrile‐3 nanofibers, the degradation activity was only reduced by 7.3% after being used five times. In addition, the simultaneous presence of organic and inorganic pollutants enhanced the removal performance overall, with organic pollutants playing a crucial role as hole scavengers. This study reveals a disruptive approach of piezo‐photocatalysis to eliminate both inorganic and organic pollutants from the environment.^[^ ^98^ ^]^

Recently, microplastic contamination is among the most serious environmental problems, with implications of significant risk for ecosystems. Figure 9E reports a novel piezo‐photocatalyst Fe_1−x_S/FeMoO_4_/MoS_2_ designed for efficient decomposition of polystyrene microplastic particles by a Fenton process. The void sea urchin‐like structure of Fe_1−x_S/FeMoO_4_/MoS_2_ promotes the electron migration being distributed on the special interpenetrating nanorods, which generates a strongly built‐in electric field. After only 30 h, the degradation efficiency of the polystyrene microplastics has reached as high as 58.46%, which is over 2.2 times than that of the Fe_1−x_S/MoS_2_ heterostructure and surpasses numerous piezoelectric photocatalysts.^[^ ^123^ ^]^

**Figure 10:**
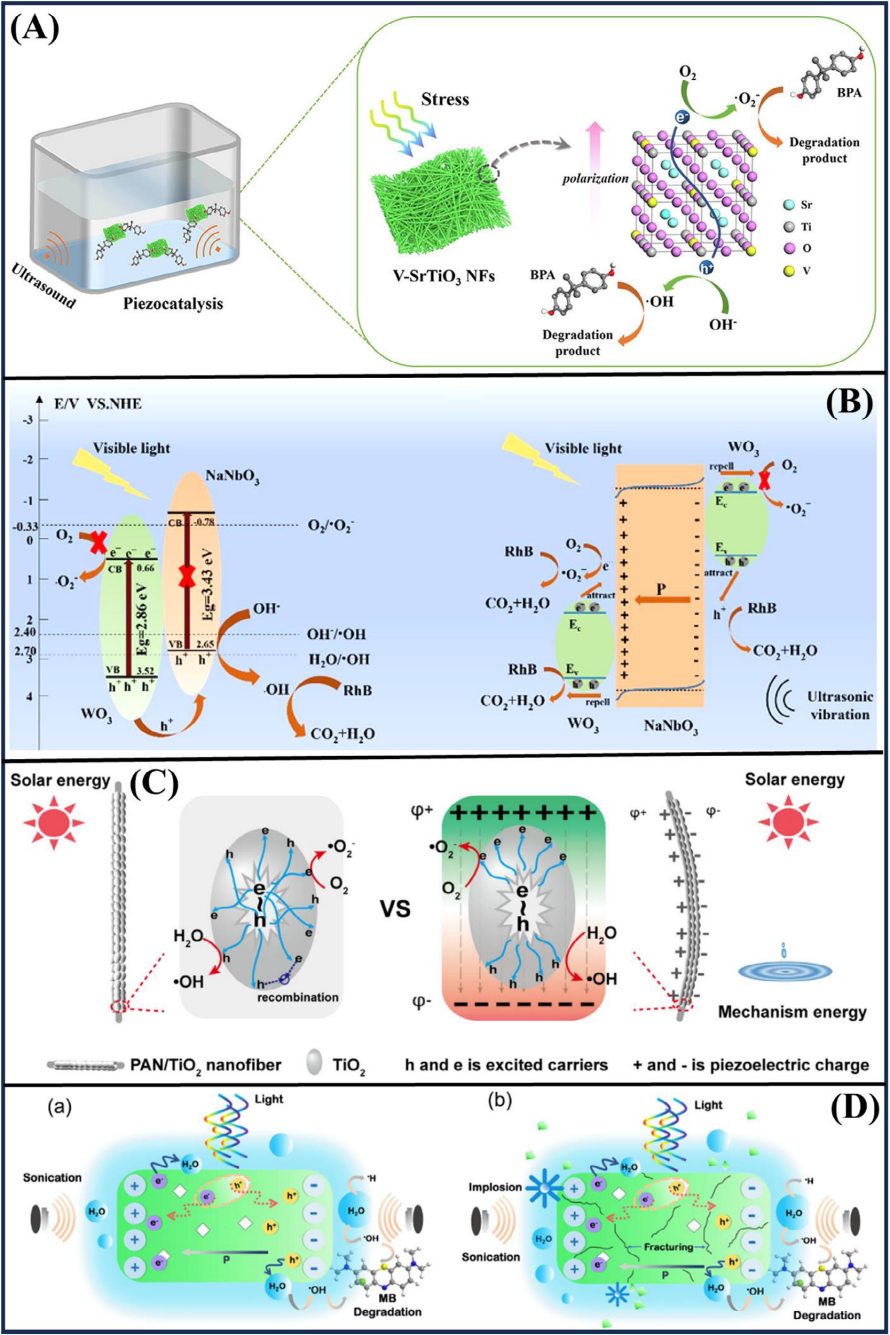
Various piezo‐photocatalytic composites for pollutant degradation, such as (A) V‐doped SrTiO_3_,^[^ ^52^ ^]^ (B) NaNbO_3_/WO_3_,^[^ ^80^ ^]^ (C) polyacrylonitrile/TiO_2_,^[^ ^124^ ^]^ (D) BiVO_4_.^[^ ^125^ ^]^ Reproduced with permission.^[^ ^52^ ^]^ Copyright 2022, Elsevier. Reproduced with permission.^[^ ^80^ ^]^ Copyright 2023, Elsevier. Reproduced with permission.^[^ ^124^ ^]^ Copyright 2022, Elsevier. Reproduced with permission.^[^ ^125^ ^]^ Copyright 2022, American Chemical Society.

An innovative approach was established for the elimination of low‐concentration bisphenol A (BPA) in water with V‐doped SrTiO_3_ by piezo‐catalytic degradation. **Figure** **10**A shows an approach that changes mechanical vibrations to chemical energy using ultrasounds. The best V‐doped SrTiO_3_ nanofibers (0.5% vanadium) reached 100% BPA removal in about 24 min, owing to the locally enhanced piezoelectricity of SrTiO_3_ and increased local asymmetry from vanadium replacement. The effects of ultrasonic power, pH, BPA concentration, and coexisting inorganic anions on decomposition were investigated. This approach offers a new promising option for degrading phenolic pollutants, and also demonstrates the application potential of the perovskite‐based nano‐piezoelectric materials in response to the issue of environmental remediation.^[^ ^52^ ^]^

Increasing the separation efficiency of photo‐produced charge carriers is the key to promoting photocatalytic activity. Figure 10B shows a novel NaNbO_3_/WO_3_ composite photocatalyst, which can efficiently improve the photocatalytic performance by combining the effects of visible light and ultrasonic vibration. Under piezo‐photocatalysis, the 5% NaNbO_3_/WO_3_ composite exhibits an exciting 73.7% of degradation toward RhB within 120 min, which is 30.1% higher than the pure photocatalysis method, where the reaction rate constant enhances 2.23 times. The results of quench experiments demonstrate that the hole (h^+^) is the dominant species in the procedures. The boosted activity can be attributed to the piezoelectric field produced originating from bending distortion of the NaNbO_3_ nanowires to promote effective charge carrier separation and many redox‐active sites to further enhance the photoconversion performance.^[^ ^80^ ^]^

Polyacrylonitrile (PAN)‐based piezoelectric nanofibers are combined with TiO_2_. The results indicate that deformation‐induced piezoelectric fields can promote the separation of photoexcited electron‐hole pairs by multiple orders of magnitude, thereby enhancing the oxidation of pollutants. Figure 10 C illustrates that the catalytic performance of PAN/TiO_2_ nanofibers in RhB degradation can be improved by a factor of 2.5 due to vibration‐induced piezoelectricity. This improvement is attributed to the efficient separation of electrons and holes within TiO_2_ nanoparticles. This new approach not only maximizes the efficiency of nanofiber mats but also harnesses renewable energy resources.^[^ ^124^ ^]^ Figure 10D demonstrates that the BiVO_4_ nanorods, prepared through a hydrothermal method, exhibit remarkable photo‐galvanic, piezo‐galvanic, and piezo‐photo‐galvanic activity, particularly under ultrasonic (35 kHz) and visible light. The BiVO_4_ nanorods show a broad range of visible‐near‐infrared absorption and contain high levels of oxygen defects, which accelerate the degradation of organic pollutants. The authors achieved 97.63% and 97.13% degradation of MB within 40 min in the piezo‐catalytic process due to the influence of ultrasound vibrations.^[^ ^125^ ^]^

## 8 Dual‐Function Piezo‐Photocatalytic Systems

**Figure 11:**
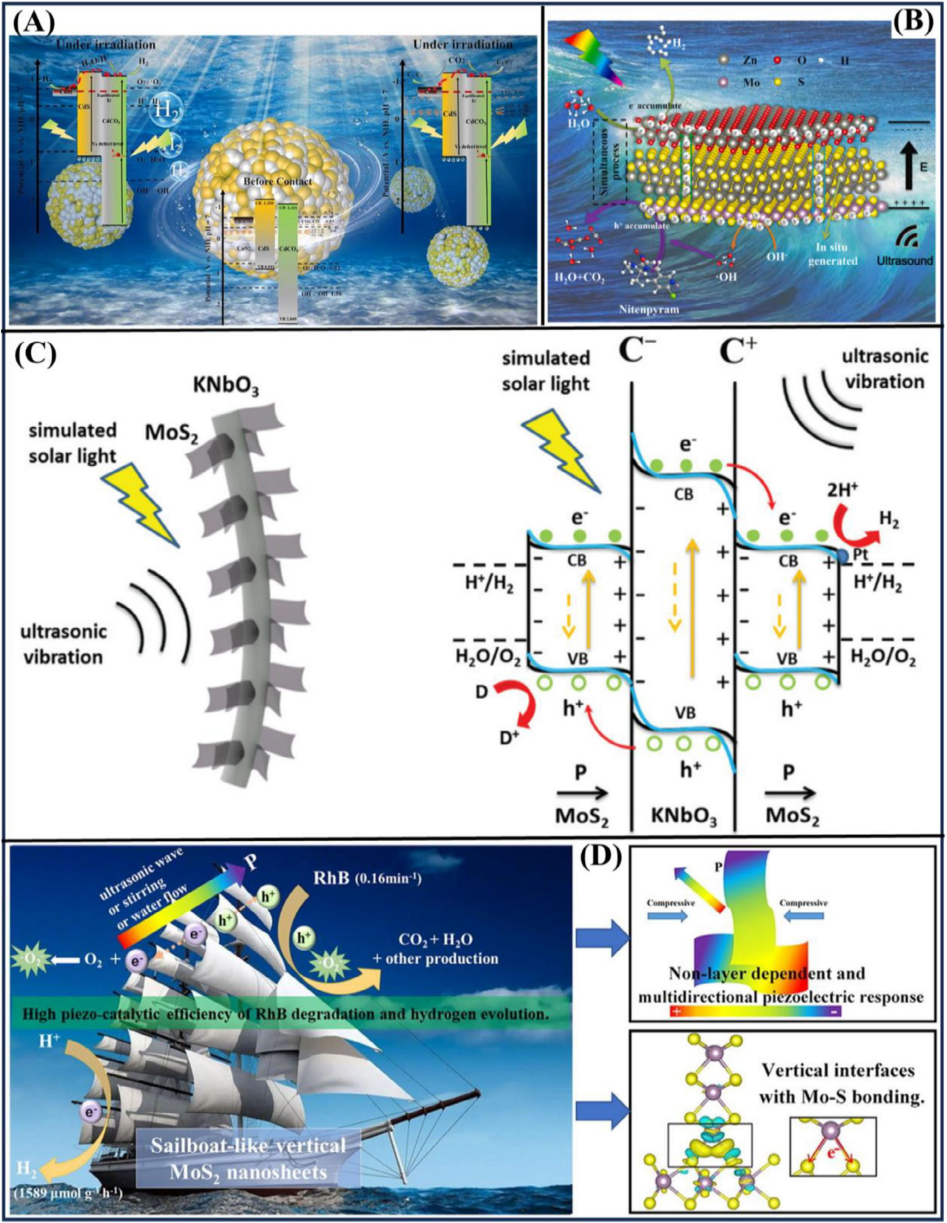
Various dual‐function piezo‐photocatalytic systems, such as (A) CdS/CdCO_3_‐CoS_2_ assisting for H_2_ evolution and CO_2_ reduction,^[^ ^78^ ^]^ (B) ZnO/MoS_2_ supporting for H_2_ recovery in wastewater treatment,^[^ ^7^ ^]^ (C) KNbO_3_/MoS_2_ using for H_2_ evolution and organic pollutant degradation,^[^ ^126^ ^]^ ((D) vertical MoS_2_ nanosheets employing for H_2_ evolution and dye degradation.^[^ ^4^ ^]^ Reproduced with permission.^[^ ^78^ ^]^ Copyright 2023, Elsevier. Reproduced with permission.^[^ ^7^ ^]^ Copyright 2024, Elsevier. Reproduced with permission.^[^ ^126^ ^]^ Copyright 2019, Royal Society of Chemistry. Reproduced with permission.^[^ ^4^ ^]^ Copyright 2023, Elsevier.

**Figure** **11**A introduces a defect‐rich CdS/CdCO_3_‐CoS_2_ photocatalyst, with the best‐performing configuration presenting a H_2_ evolution rate that reached values of 64 867.88 µmol·g^−1^·h^−1^, which is higher than those of CoS_2_‐CdS and pure CdS by factors of 3.23 and 49.45, respectively. Furthermore, the authors attained a high CO_2_ reduction rate of 654.7 µmol·g^−1^·h^−1^. The shell‐yolk architecture optimizes light multi‐scattering and results in a hydrogen evolution rate that is 1.52 times greater compared to a standard hollow structure. The significant notch depth is also enhanced by the Mie resonant effects and the generation of defect energy levels in CdCO_3_, creating a strong Schottky‐type/type II heterojunction. This work opens a new avenue for understanding oxygen vacancies and for interpreting photocatalytic mechanisms.^[^ ^78^ ^]^ Figure 11B presents a new ZnO/MoS_2_ piezo‐photocatalytic‐based H_2_ energy system for significantly increasing the separation and transfer efficiency of photoproduced electrons and holes. This resulted in significant decomposition of nitenpyram in only 60 min, with a remarkable H_2_ generation activity of 746.56 µmol·g^−1^·h^−1^. These tuning changes electron transfer ways, and most electrons flow toward ZnO for H_2_ evolution, and holes accumulate in MoS_2_ for producing hydroxyl radicals. This enhanced coupling greatly promotes the degradation of the contaminants and H_2_ production. This piezo‐photocatalytic system is not only the first to realize the reuse of H_2_ for treating wastewater by a heterostructure, but also represents a new methodology to develop green catalytic systems.^[^ ^7^ ^]^

Figure 11C illustrates a hetero‐structured photocatalyst with piezoelectric KNbO_3_ nanowires and few‐layer MoS_2_ nanosheets via an easy two‐step hydrothermal route. The as‐obtained KNbO_3_/MoS_2_ heterostructures exhibited good performance in photocatalytic H_2_ generation and decomposition of an organic pollutant dye of RhB under artificial solar irradiation, which was well beyond that of the pure components. Harnessing the solar and mechanical energies by relying on the ultrasonic vibrations resulted in exceptional photocatalytic activity, due to superior charge separation of the heterojunction and the piezoelectric field. These discoveries pave the way for emerging novel nanocomposites with an effective piezoelectric material, which in turn provide exciting opportunities for cleaning the environment and sustainable energy applications.^[^ ^126^ ^]^

By wrinkle‐released method, the sailboat‐like MoS_2_ nanosheets, which are vertically‐oriented MoS_2_ nanosheets on one side and are sitting on the horizontal substrate elsewhere, could be fabricated as shown in Figure 11D. This architecture offers numerous vertical interfaces and potential control over high‐resolution phase composition, reinforcing the mechanical energy harvesting capability. The significant improvements in both in‐plane and out‐of‐plane polarization led to a higher piezo‐response and a larger piezo‐potential magnitude caused by the active edge sites of nanosheets. The Mo‐S bonded contacts additionally promote the separation between electrons and holes. Significantly, the piezo‐degradation rate of RhB and the H_2_ evolution rate under ultrasonic stirring are as high as 0.16 min^−1^ and 1598 µmol·g^−1^·h^−1^ for nanosheets, which are even superior to that of few‐layer MoS_2_ nanosheets. The authors could degrade up to 94% RhB (500 mL) within 60 min. The results also suggest the great potential of the sailboat‐like MoS_2_ nanosheets for novel practices in green energy production, clean environmental technologies, and unique materials applications, which might stimulate considerable breakthroughs in these subjects.^[^ ^4^ ^]^

**Figure 12:**
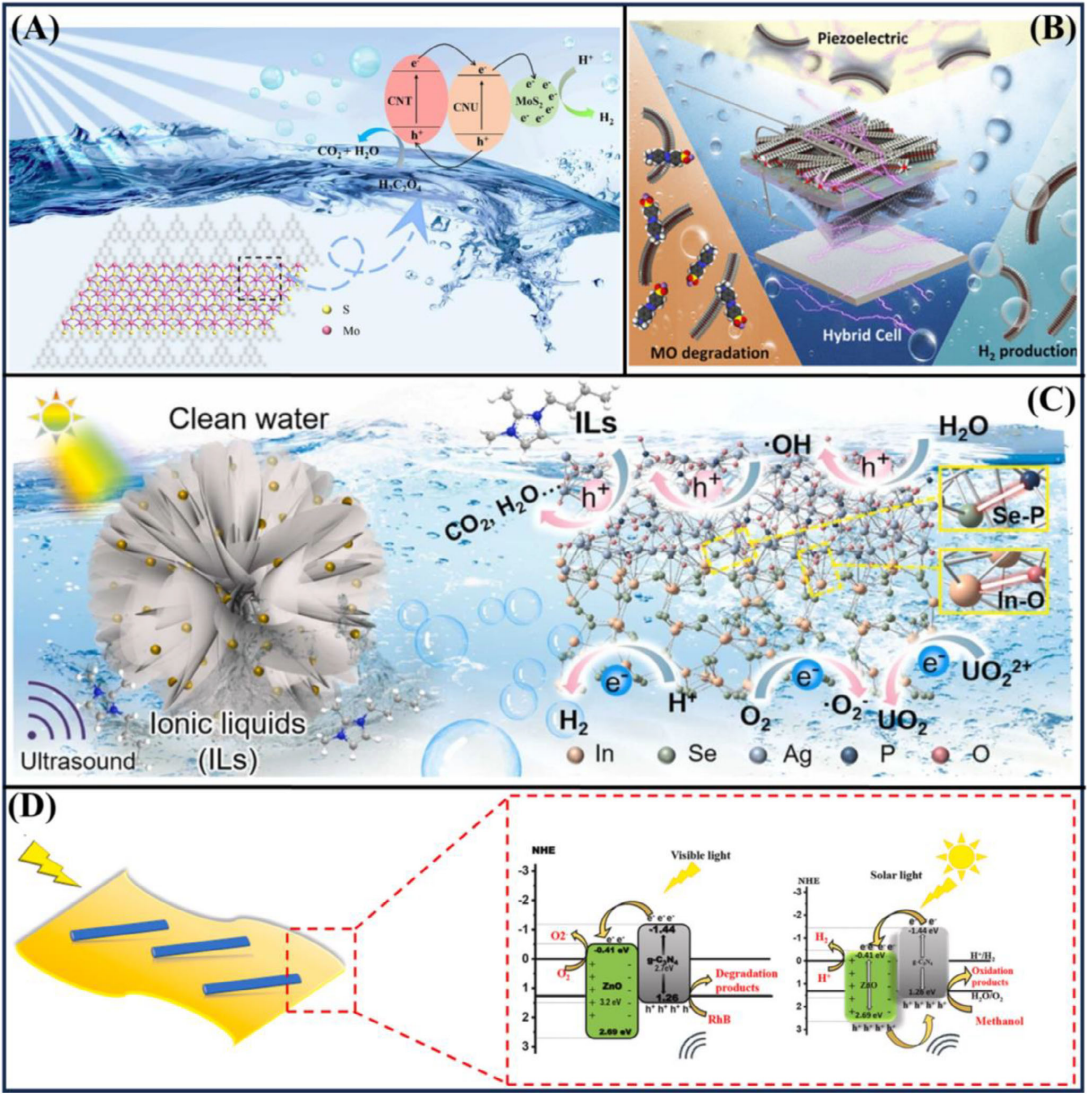
A) Simultaneous photocatalytic H_2_ evolution and wastewater purification via 1T/2H‐MoS2@CNT/CNU.^[^ ^127^ ^]^ Various dual‐function piezo‐photocatalytic materials, such as (B) hybrid cell employing for H_2_ generator and water treatment,^[^ ^128^ ^]^ (C) In_2_Se_3_@Ag_3_PO_4_ for synergistic environmental remediation, H_2_ evolution, and uranium reduction,^[^ ^77^ ^]^ (D) g‐C_3_N_4_/ZnO enhancing for H_2_ evolution and pollutant removal.^[^ ^129^ ^]^ Reproduced with permission.^[^ ^127^ ^]^ Copyright 2021, Elsevier. Reproduced with permission.^[^ ^128^ ^]^ Copyright 2015, American Chemical Society. Reproduced with permission.^[^ ^77^ ^]^ Copyright 2025, Elsevier. Reproduced with permission.^[^ ^129^ ^]^ Copyright 2023, Elsevier.

The exploration of affordable earth‐abundant element‐based photocatalysts for producing H_2_ from wastewater is a very favorable way of addressing the world's energy and environmental issues. As shown in **Figure** **12**A, the authors fabricated flower‐like 1T/2H‐MoS_2_ anchored with g‐C_3_N_4_ (derived from thiourea and urea), denoted as CNT/CNU, using a simple ultrasonic dispersant method. The photocatalytic activity of these heterojunction photocatalysts was examined in simulated wastewater containing oxalic acid (OA). Notably, the H_2_ release rate of 20% 1T/2H‐MoS_2_@CNT/CNU reached 1563.32 µmol·g^−1^·h^−1^, which was 96 times higher than that of CNT/CNU alone, and the total organic carbon (TOC) removal rate was 67.5%. This enhanced performance can be attributed to a novel double‐interface architecture that optimizes charge separation. The flower‐like structure provides large active sites and increases the ability to absorb visible light. This study suggests that the added cocatalysts enhance charge transfer, offering a new prospect for utilizing wastewater as a sustainable source to produce clean energy.^[^ ^127^ ^]^ Also, Figure 12B shows a new advancement in energy harvesting that combines two sources of energy to drive photocatalytic reactions. This hybrid cell harvests energy from light and vibrations, achieving power‐free photocatalysis. It features a novel metal‐semiconductor branched heterostructure that effectively absorbs visible light and harnesses piezoelectricity. The hierarchical structure of the conductive flexible piezo‐electrode enables strong light scattering. This self‐sustained photocatalytic system can significantly contribute to H_2_ production and water treatment, bringing us closer to a sustainable future with abundant clean energy and water resources.^[^ ^128^ ^]^

Recently, ionic liquids (ILs) have become significant contaminants in a water‐based ecosystem due to their intrinsic toxicity. As shown in Figure 12C, the authors reported the In_2_Se_3_@Ag_3_PO_4_ S‐scheme piezo‐photocatalyst, which features In‐O and Se‐P covalent bonds for superior degradation of 1‐butyl‐2,3‐dimethylimidazole bromide (BMMIm‐Br). This material demonstrates an impressive degradation efficiency of 98.3% through piezo‐photocatalysis. BMMIm‐Br can also act as a complete sacrificial reagent, producing 582.7 µmol·g^−1^·h^−1^ of hydrogen and achieving a removal rate of 97.2% for U(VI) heavy metal ions. Overall, toxicity testing shows the diminished toxicity of these degradative intermediates, spotlighting the promising applications of this research in addressing environmental pollution issues.^[^ ^77^ ^]^ In addition, Figure 12D illustrates that g‐C_3_N_4_/ZnO nanocomposites show excellent piezo‐photocatalytic activity for inducing RhB degradation and H_2_ generation. Remarkably, the urea‐derived composite g‐C_3_N_4_‐U‐/ZnO surpasses all the composites synthesized from melamine, thiourea, and dicyanamide, possessing the highest surface area of 182.3 m^2^ g^−1^. The H_2_ evolution rate and dye decomposition show incredible results of 827.5 µmol·g^−1^·h^−1^ and 82%. By incorporating ultrasounds into the photocatalytic reactions, that value increased to 1497.5 µmol·g^−1^·h^−1^ of hydrogen and nearly complete dye degradation (an impressive 99%) due to the strong superoxide radicals formed. Furthermore, the g‐C_3_N_4_‐U‐/ZnO composite exhibited good stability after four cycles, indicating positive efficiency. By combining piezo‐catalysis and photocatalysis, g‐C_3_N_4_‐U‐/ZnO emerges as a promising material for sustainable energy conversion and environmental remediation.^[^ ^129^ ^]^

Dual‐function piezo‐photocatalytic systems represent a groundbreaking advancement that seamlessly combines the unique features of photocatalysis and piezo‐catalysis within a coherent and synergistic system. This exceptional combination not only enhances catalytic activity but also enables a wide range of interactions, which, to some extent, broadens the potential applications of these systems in a variety of fields such as environmental cleanup, water treatment, and energy transformation devices (e.g., H_2_ evolution).

## 9 Challenges and Future Perspectives

Piezo‐photocatalysis has made rapid progress since its inception, owing to its high catalytic efficiency and easy of accessibility. However, several core concerns must be addressed for further development. From a materials perspective, the complexity and potential of piezo‐photocatalysts are determined by either semiconductors or insulators.^[^ ^130^ ^]^ It is worth examining whether a single substitution leads to multiform impacts on piezo‐catalytic efficiency.^[^ ^131^ ^]^ While dopants can enhance the piezoelectric constant, it can also alter the band structure, which requires careful consideration.^[^ ^132^ ^]^ Additionally, piezoelectricity can be affected by processes such as annealing, which can lead to the production of oxygen vacancies on surfaces.^[^ ^86^ ^]^ The combined or competing effects of other factors on catalytic activities also need to be considered, as this has not been thoroughly investigated. Furthermore, the piezo‐photocatalysts reported thus far are not ready for real applications. Their low performance in mechanical energy harvesting and poor recycling ability significantly impede their broader application under practical conditions. Regarding the mechanism, the relationship between the semiconductor and the piezoelectric charge characteristics remains poorly understood.^[^ ^133^ ^]^

Understanding the connection between free charge carrier concentrations and redox thermodynamics in integrated piezo‐photocatalysts is crucial.^[^ ^134^ ^]^ Addressing these key issues and pursuing these directions will undoubtedly advance the field of piezo‐photocatalysis, bringing us closer to realizing the significant impact of these new piezo‐photocatalytic forms on environmental remediation,^[^ ^135^ ^]^ energy,^[^ ^136^ ^]^ and environment‐related applications.^[^ ^137^ ^]^ First, the sensitivity of piezo‐photocatalysts with respect to mechanical stimulation needs further improvement to capture more minute change in energy.^[^ ^138^ ^]^ Most piezo‐photocatalytic reactions have been performed in the presence of intense ultrasonics, which rarely occur in natural habitats. For instance, dye pollutants can be removed using piezoelectric‐catalytic porous PANI/(Ba_0.85_Ca_0.15_)(Zr_0.1_Ti_0.9_)O_3_/PVDF at low frequencies and under natural conditions.^[^ ^139^ ^]^ Second, to gain a better understanding of the corresponding action mechanisms, more theoretical work is still necessary regarding the electronic structure and charge transfer.^[^ ^140^ ^]^ For instance, S‐scheme heterojunction as piezo‐photocatalytic oxygen vacancy‐rich Bi_2_MoO_6_‐SOVs/MgFe_2_O_4_ have been synthesized, and applied a calculated theory for efficient norfloxacin degradation.^[^ ^107^ ^]^ Finally, piezo‐photocatalysts are currently limited to applications in pollutant degradation and water splitting. There are high expectations for the development of these applications in other areas, such as nitrogen fixation, selective organic synthesis, and medical treatments.^[^ ^141^ ^]^ For example, cooperating rhombohedral and orthorhombic ZnSnO_3_ boost the catalytic efficiency performance of the degradation of dye, and the conversion of N_2_ to NH_3_ via piezo‐catalysis and piezo‐photocatalysis.^[^ ^142^ ^]^ Additionally, there is an increasing demand for enhancing the stability and recyclability of piezo‐photocatalysts.^[^ ^143^ ^]^ Furthermore, 3D printing, also known as additive manufacturing, is a technique in which 3D objects are built from digital models by adding material, layer by layer. The combination of 3D printing and catalysts is a novel trend attracting many researchers. For example, to enhance interfacial charge transfer, 3D‐printed Mo_2_CT_x_‐UiO‐66@rGQDs composites and semiconducting BiVO_4_ have been successfully integrated for the photocatalytic degradation of atrazine. Impressive results were obtained, with a 98.19% atrazine degradation rate over 120 min, corresponding to a 0.03135 min^−1^ reaction rate.^[^ ^144^ ^]^ Given the successful combination of 3D printing and photocatalysis, future research should explore the integration of 3D printing with piezo‐photocatalysis. It is inspiring to witness the advancement of highly instrumented, flexible devices with multi‐irradiation source‐driven, efficient catalytic activity, ranging from solar light to weak forces generated by human activities. These developments provide an optimistic outlook for addressing environmental and energy challenges.

Finally, several issues with the industrial use of piezoelectric materials include low energy density, temperature sensitivity, manufacturing challenges, limited application options, and concerns over long‐term reliability. While these materials are useful for some small‐scale applications like sensing, they face difficulties for large‐scale energy harvesting or high‐precision industrial uses because of their inherent limitations.

## 10 Conclusion

**Figure 13:**
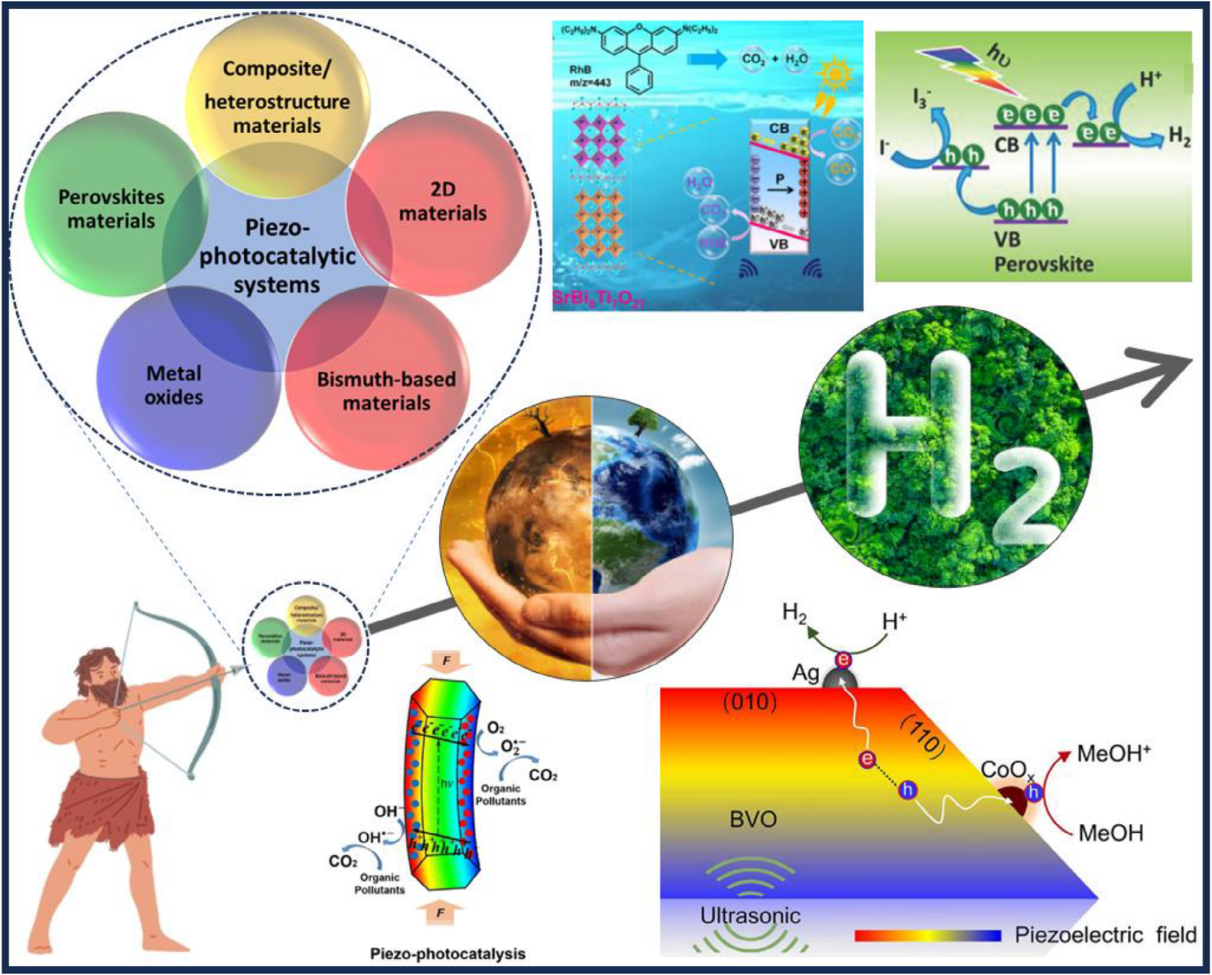
Achieve two aims at once: synergistic environmental remediation and sustainable H_2_ evolution through dual‐function piezo‐photocatalytic systems.

In this work, recent developments in piezoelectric materials used for sustainable H_2_ evolution and environmental remediation (e.g., CO_2_ reduction, degradation of organic pollutants, wastewater treatment, etc.) are reviewed (as shown in **Table** **3** and **Figure** **13**). The authors start with a summary of the piezoelectric principles, material structures, and performance factors driving the functionality of these materials. The authors categorize classical piezoelectric materials capable of H_2_ evolution, CO_2_ reduction, and pollutant degradation, emphasizing dual‐function piezo‐photocatalytic systems, such as heterostructure composites and surface dopants, which enhance performance. One leading application is their use in H_2_ evolution and environmental remediation. However, the range of applications is often limited by the bandwidth and efficiency of traditional piezoelectric transducers. The practical use of piezoelectric materials is constrained by factors such as material costs, energy requirements, durability, material properties, integration, and environmental issues. Although these materials show promise for niche applications, pollutants and related concerns have restricted their widespread adoption, especially in high‐volume systems. Despite challenges like low driving force and incomplete degradation, the advancement of piezoelectric catalysis remains promising. Future work in this field can be enhanced by developing highly efficient catalysts, promoting material design, and employing various characterization tools and theoretical calculations to discover new applications. Exciting developments are anticipated in the area of research on heterostructures, which have demonstrated inotably increased photocatalytic activity for the decomposition of chemical pollutants and H_2_ production compared to their components. This enhanced performance results from the piezoelectric fields generated by the piezo‐photocatalytic effect in heterostructure catalysts, along with the built‐in electric field originating from their heterojunctions. These mechanisms aid in the separation of photo‐generated electron‐hole pairs. The findings of this study may provide meaningful guidance for designing high‐efficiency piezo‐photocatalysts for H_2_ evolution and environmental pollution control in the future.

**Table 3:** Summary of literature review related to the piezo‐photocatalytic systems.

Platforms	Applications/Issues	Reference
Photo‐/electro‐/piezo‐catalytic	Pollutants elimination	[5]
Hybrid piezo‐photocatalysts	Utilizing dual functionalities to enhance charge dynamics and improve catalytic performance	[10]
Piezo‐catalysis and piezo‐photocatalysis	Oxygen activation, water decontamination, water splitting, sterilization, cell treatment, polymerization, other organic reactions	[11]
2D transition metal dichalcogenides	Synthesis methods, role in catalytic processes	[17]
Piezo‐photocatalytic materials	CO_2_ reduction	[145]
Piezoelectric materials	Pollutants degradation	[146]
g‑C_3_N_4_‑based composites	Photocatalytic H_2_ evolution	[147]
Graphene‐based materials	Metronidazole degradation	[148]
Graphene‐based catalysts	Mitigation of environmentally hazardous pollutants	[38]
Piezoelectric materials	H_2_ evolution, environmental remediation	This work

## Conflict of Interest

The authors declare no conflict of interest.

## References

[1] A. Fazli , A. Athanassiou , D. Fragouli , Adv. Sci. (Weinh) 2025, 12, 04352. 10.1002/advs.202504352 PMC12376683 40557804

[2] S. Asgari , G. Mohammadi Ziarani , A. Badiei , Y. Vasseghian , Chem. Eng. J. 2024, 487, 150600.

[3] C. Bi , H. Xu , G. Zhou , H. Song , J. Xu , Q. Li , B. Wang , P. Ding , L. Li , P. K. Chu , H. Xu , X. Zhu , J. Ding , Chem. Eng. J. 2024, 501, 157345.

[4] Y. Wang , H. Ma , J. Liu , Z. Zhang , Y. Yu , S. Zuo , J. Colloid Interface Sci. 2023, 642, 304. 37011449 10.1016/j.jcis.2023.03.159

[5] S. Wang , Y. Li , Q. Liu , J. Wang , Y. Zhao , Y. Cai , H. Li , Z. Chen , J. Photochem. Photobiol. A, Chem. 2023, 437, 114435.

[6] Y. Jiang , J. Xie , Z. Lu , J. Hu , A. Hao , Y. Cao , J. Colloid Interface Sci. 2022, 612, 111. 34983011 10.1016/j.jcis.2021.10.170

[7] X. Dai , Z. Liu , Z. Zhao , J. Wang , H. Luo , L. Cheng , W. Chen , J. Cleaner Prod. 2024, 447, 141540.

[8] H. Hu , X. Li , K. Zhang , G. Yan , W. Kong , A. Qin , Y. Ma , A. Li , K. Wang , H. Huang , X. Sun , T. Ma , Adv. Mater. 2025, 37, 2419023. 40159815 10.1002/adma.202419023 PMC12087703

[9] W. Jiang , H. Zhu , J. Yang , B. Q. L. Low , W. Y. Wu , M. Chen , J. Ma , R. Long , J. Low , H. Zhu , J. Z. X. Heng , K. Y. Tang , C. H. T. Chai , M. Lin , Q. Zhu , Y. W. Zhang , D. Chi , Z. Li , X. J. Loh , Y. Xiong , E. Ye , Adv. Sci. (Weinh) 2023, 10, 2303448. 37544890 10.1002/advs.202303448 PMC10558689

[10] X. Yan , J. Zhu , J. Xu , S. Yang , Y. Liu , H. He , S. Liu , K. Wang , Chem. Eng. J. 2024, 500, 157440.

[11] S. Tu , Y. Guo , Y. Zhang , C. Hu , T. Zhang , T. Ma , H. Huang , Adv. Funct. Mater. 2020, 30, 2005158.

[12] R. Mohanty , S. Mansingh , K. Parida , K. Parida , Mater. Horiz. 2022, 9, 1332. 35139141 10.1039/d1mh01899j

[13] D. Masekela , S. A. Balogun , T. L. Yusuf , S. Makgato , K. D. Modibane , J. Water Process Eng. 2025, 71, 107172.

[14] S. Zhang , Z. Liu , M. Ruan , Z. Guo , E. Lei , W. Zhao , D. Zhao , X. Wu , D. Chen , Appl. Catal., B 2025, 262, 2020.

[15] X. Liu , L. Xiao , Y. Zhang , H. Sun , J. Materiomics 2020, 6, 256.

[16] G. Yang , Q. Chen , W. Wang , S. Wu , B. Gao , Y. Xu , Z. Chen , S. Zhong , J. Chen , S. Bai , ACS Appl Mater Interfaces 2021, 13, 15305. 33775098 10.1021/acsami.1c01550

[17] D. Thakur , C. Porwal , V. Singh Chauhan , V. Balakrishnan , Sep. Purif. Technol. 2024, 337, 126462.

[18] Y. Zhang , Y. Wang , W. Xu , Y. Wu , C. Zeng , Y. Wang , W. Zhong , R. Yang , Chem. Eng. J. 2024, 431, 2022.

[19] R. Lei , F. Gao , J. Yuan , C. Jiang , X. Fu , W. Feng , P. Liu , Appl. Surf. Sci. 2022, 576, 151851.

[20] X. Xu , L. Xiao , Z. Wu , Y. Jia , X. Ye , F. Wang , B. Yuan , Y. Yu , H. Huang , G. Zou , Nano Energy 2020, 78, 105351.

[21] M. Wang , Y. Zuo , J. Wang , Y. Wang , X. Shen , B. Qiu , L. Cai , F. Zhou , S. P. Lau , Y. Chai , Adv. Energy Mater. 2019, 9, 1901801.

[22] X. Ma , Y. Gao , B. Yang , X. Lou , J. Huang , L. Ma , D. Jing , Mater. Today Nano 2023, 21, 100289.

[23] K. Zhou , W. Liu , P. Wang , J. Chen , H. Che , B. Liu , Chem. Eng. J. 2024, 480, 148012.

[24] S. Zhong , Y. Wang , Y. Chen , X. Jiang , M. Lin , C. Lin , T. Lin , M. Gao , C. Zhao , X. Wu , Chem. Eng. J. 2024, 488, 151002.

[25] X. Sun , K. Lv , F. Liu , P. Wang , K. Zhang , J. Zhang , P. Chen , Chem. Eng. J. 2024, 497, 154504.

[26] J. Yuan , X. Huang , L. Zhang , F. Gao , R. Lei , C. Jiang , W. Feng , P. Liu , Appl. Catal., B 2024, 278, 2020.

[27] S. Lin , S. Li , H. Huang , H. Yu , Y. Zhang , Small 2022, 18, 2106420. 10.1002/smll.202106420 34936197

[28] C. H. Chiang , C. C. Lin , Y. C. Lin , C. Y. Huang , C. H. Lin , Y. J. Chen , T. R. Ko , H. L. Wu , W. Y. Tzeng , S. Z. Ho , Y. C. Chen , C. H. Ho , C. J. Yang , Z. W. Cyue , C. L. Dong , C. W. Luo , C. C. Chen , C. W. Chen , J. Am. Chem. Soc. 2024, 146, 23278. 39049154 10.1021/jacs.4c05798 PMC11345765

[29] Z. Guo , X. Li , X. Zhou , J. Meng , Y. Xia , X. Wang , Y. Li , J. Water Process Eng. 2025, 71, 107156.

[30] D. Li , Q. Wen , C. Gao , Y. Zhang , J. Zhou , S. Liu , F. Song , K. Wang , Chem. Eng. J. 2025, 498, 2024.

[31] S. Chang , H. Gu , H. Zhang , X. Wang , Q. Li , Y. Cui , W. L. Dai , J. Colloid Interface Sci. 2023, 644, 304. 37120879 10.1016/j.jcis.2023.04.111

[32] N. Li , G. Zhao , Y. Wu , Y. Li , K. Zhao , W. Fu , S. Zhang , J. Ma , ACS Appl. Nano Mater. 2024, 7, 26952.

[33] J. Hu , R. Zhao , J. Ni , W. Luo , H. Yu , H. Huang , B. Wu , Y. Wang , J. Han , R. Guo , Adv. Sci. (Weinh) 2024, 11, 2410357. 39413017 10.1002/advs.202410357 PMC11615802

[34] W. Liu , P. Wang , Y. Ao , J. Chen , X. Gao , B. Jia , T. Ma , Adv. Mater. 2022, 34, 2202508. 10.1002/adma.202202508 35560713

[35] J. Liu , W. Qi , M. Xu , T. Thomas , S. Liu , M. Yang , Angew. Chem. Int. Ed. Engl. 2023, 62, 202213927. 10.1002/anie.202213927 36316280

[36] T. Jiang , Y. Wang , C. Cai , C. Nie , H. Peng , Z. Ao , Environ. Sci. Ecotechnol. 2025, 23, 100495.

[37] P. Dhiman , J. Sharma , A. Kumar , G. Sharma , G. Rana , Mater. Today Sustain. 2024, 28, 100973.

[38] Y. Vasseghian , V. T. Le , S.‐W. Joo , E.‐N. Dragoi , H. Kamyab , S. Chelliapan , J. Cleaner Prod. 2022, 365, 132702.

[39] J. Li , X. Liu , G. Zhao , Z. Liu , Y. Cai , S. Wang , C. Shen , B. Hu , X. Wang , Sci. Total Environ. 2023, 869, 161767. 36702283 10.1016/j.scitotenv.2023.161767

[40] W. Dong , H. Xiao , Y. Jia , L. Chen , H. Geng , S. U. H. Bakhtiar , Q. Fu , Y. Guo , Adv. Sci. (Weinh) 2022, 9, 2105368. 35240724 10.1002/advs.202105368 PMC9069204

[41] N. Meng , W. Liu , R. Jiang , Y. Zhang , S. Dunn , J. Wu , H. Yan , Prog. Mater. Sci. 2023, 138, 101161.

[42] N. Sezer , M. Koç , Nano Energy 2021, 80, 105567.

[43] X. Mo , M. Wang , H. Song , J. Li , Z. Wu , Z. Lin , Chem. Eng. J. 2024, 498, 155105.

[44] J. Wu , H. Huang , C. Zhang , H. Shi , Y. Chen , Chem. Eng. J. 2025, 508, 161137.

[45] M. Venkatesan , W.‐C. Lin , W.‐C. Chen , J. Chandrasekar , Y.‐H. Huang , K.‐W. Lin , Z.‐S. Syu , J.‐H. Lin , W.‐H. Chiang , W.‐R. Liu , Y. Zhou , C.‐C. Kuo , Chem. Eng. J. 2025, 505, 159541.

[46] Z. Liang , C.‐F. Yan , S. Rtimi , J. Bandara , Appl. Catal., B 2019, 241, 256.

[47] X. Sun , J. Zhang , J. Ma , T. Xian , G. Liu , H. Yang , Chem. Eng. J. 2024, 496, 153961.

[48] L. Yang , J. Guo , S. Chen , A. Li , J. Tang , N. Guo , J. Yang , Z. Zhang , J. Zhou , J. Colloid Interface Sci. 2024, 659, 776. 38215614 10.1016/j.jcis.2024.01.022

[49] D. Takhar , B. Birajdar , R. K. Ghosh , Phys. Chem. Chem. Phys. 2024, 26, 16261. 38804603 10.1039/d4cp01047g

[50] C. Du , S. Nie , C. Zhang , T. Wang , S. Wang , J. Zhang , C. Yu , Z. Lu , S. Dong , J. Feng , H. Liu , J. Sun , J. Colloid Interface Sci. 2022, 606, 1715. 34500170 10.1016/j.jcis.2021.08.152

[51] A. M. AlAhzm , M. O. Alejli , D. Ponnamma , Y. Elgawady , M. A. A. Al‐Maadeed , J. Mater. Sci., Mater. Electron. 2021, 32, 14610.

[52] Q. Zhou , N. Li , D. Chen , Q. Xu , H. Li , J. He , J. Lu , Chem. Eng. Sci. 2022, 247, 116707.

[53] J. Wu , N. Qin , D. Bao , Nano Energy 2018, 45, 44.

[54] Y. Wang , X. Li , Y. Chen , Y. Li , Z. Liu , C. Fang , T. Wu , H. Niu , Y. Li , W. Sun , W. Tang , W. Xia , K. Song , H. Liu , W. Zhou , Adv. Mater. 2023, 35, 2305257. 10.1002/adma.202305257 37530983

[55] J. Gao , J. Xiao , S. Luo , X. Ji , C. Yin , Y. Wu , X. Zhao , Y. Wang , Adv. Sci. (Weinh) 2025, 08062. 10.1002/advs.202508062 PMC12520523 40642780

[56] Y. Wu , D. Yang , Y. Zhang , S. Jiao , W. Tang , Z. Wang , N. Wu , Y. Wang , W. Zhong , A. Zhang , J. Hao , H.‐L. Cai , X. S. Wu , Chem. Eng. J. 2022, 439, 135640.

[57] X. Zhu , H. Xu , C. Bi , H. Song , G. Zhou , K. Zhong , J. Yang , J. Yi , H. Xu , X. Wang , Ultrason. Sonochem. 2023, 101, 106653. 37918293 10.1016/j.ultsonch.2023.106653 PMC10638044

[58] Q. Tang , J. Wu , X.‐Z. Chen , R. Sanchis‐Gual , A. Veciana , C. Franco , D. Kim , I. Surin , J. Pérez‐Ramírez , M. Mattera , A. Terzopoulou , N. Qin , M. Vukomanovic , B. J. Nelson , J. Puigmartí‐Luis , S. Pané , Nano Energy 2023, 108, 108202.

[59] N. L. M. Khoa , Y. Vasseghian , S.‐W. Joo , Adv. Sustain. Syst. 2025, 9, 2500031.

[60] P. P. Ly , D.‐V. Nguyen , T. A. Luu , M. C. Nguyen , P. D. M. Phan , H. P. Toan , T. V. Nguyen , M.‐T. Pham , T. D. T. Ung , D. D. Bich , H. T. Pham , H. T. N. Nguyen , W. J. Yu , S. H. Hur , N. Q. Hung , H.‐T. Vuong , Chem. Eng. J. 2023, 504, 2025.

[61] P. Sengupta , A. Ghosal , S. Haldar , R. Ray , Chem. Eng. J. 2025, 503, 158486.

[62] T. Yang , P. Ren , X. Qi , X. Wang , Q. Meng , Z. Liu , S. Yang , Appl. Surf. Sci. 2023, 628, 157363.

[63] S. Tu , H. Huang , T. Zhang , Y. Zhang , Appl. Catal., B 2017, 219, 550.

[64] M. Wang , H. Yu , K. Yu , Chem. Eng. J. 2023, 470, 144100.

[65] S. Atri , S. Uma , R. Nagarajan , M. Gregor , T. Roch , M. F. Edelmannova , M. Reli , K. Koci , M. Motola , O. Monfort , Energy Adv. 2024, 3, 1956.

[66] M. Bartoli , A. Tagliaferro , 2024, 21, 100948.

[67] S. Perumal , W. Lee , R. Atchudan , Chemosphere 2022, 306, 135521. 35780986 10.1016/j.chemosphere.2022.135521

[68] T. G. Levitskaia , N. P. Qafoku , M. E. Bowden , R. Matthew Asmussen , E. C. Buck , V. L. Freedman , C. I. Pearce , ACS Earth Space Chem. 2022, 6, 883.

[69] J. Liu , S. Du , Q. Zhu , A. Labidi , H. Wang , C. Wang , J. Environ. Chem. Eng. 2024, 12, 114790.

[70] A. Batool , I. Kopp , M. Kubeil , M. Bachmann , P. C. Andrews , H. Stephan , Dalton Trans. 2025, 54, 5614. 40040450 10.1039/d5dt00163c

[71] J. Ghosh , P. Priyadarshini , Z. T. Younus , Q. Jia , W. Nie , J. L. MacManus‐Driscoll , R. L. Z. Hoye , MRS Energy Sustain. 2025. 10.1557/s43581-025-00142-5 PMC12572057 41180446

[72] F. Pan , B. Peerless , S. Dehnen , Acc. Chem. Res. 2023, 56, 1018. 37067095 10.1021/acs.accounts.3c00020 PMC10157893

[73] S. Yetiman , H. Peçenek , F. Kılıç Dokan , S. Sanduvaç , M. Serdar Onses , E. Yılmaz , E. Sahmetlioglu , ChemElectroChem 2024, 11, 202300819.

[74] W. P. Utomo , M. K. H. Leung , Z. Yin , H. Wu , Y. H. Ng , Adv. Funct. Mater. 2024, 32, 2021.

[75] J. Yin , C. Sun , J. Environ. Chem. Eng. 2025, 13, 119042.

[76] W. He , Q. Xiao , Z. Qiu , F. He , Q. Chen , L. Hu , H. Wang , Chem. Eng. J. 2024, 494, 152947.

[77] X. Zhang , Y. Chen , R. Guo , Z. Zhang , Z. Sun , Y. Zhang , F. Yan , Appl. Catal. B, Environ. Energy 2025, 372, 125314.

[78] S. Zhang , S. Du , Y. Wang , Z. Han , X. Li , G. Li , Q. Hu , H. Xu , Chem. Eng. J. 2025, 454, 2023.

[79] H. Yu , Y. Ji , Y. Zhang , S. Tu , S. K. Boong , H. K. Lee , F. Guo , C. Zhou , J. Han , ACS Sustain. Chem. Eng. 2024, 12, 9947.

[80] X. Yan , S. Zhang , L. Pan , T. Ai , Z. Li , Y. Niu , Inorg. Chem. Commun. 2023, 158, 111510.

[81] S. Xu , C. Bao , M. Al Mahadi Hasan , X. Zhang , C. Li , Y. Yang , Nano Energy 2024, 127, 109720.

[82] B. Erim , Z. Cigeroglu , S. Sahin , Y. Vasseghian , Chemosphere 2022, 291, 132929. 34800511 10.1016/j.chemosphere.2021.132929

[83] L. Zhu , W. Gu , H. Li , W. Zou , H. Liu , Y. Zhang , Q. Wu , Z. Fu , Y. Lu , Appl. Surf. Sci. 2020, 528, 146837.

[84] X. Zheng , L. Yang , Y. Li , L. Yang , S. Luo , Electrochim. Acta 2019, 298, 663.

[85] Y. Jiang , C. Ying , T. Sajjad , S. Mofarah , C. Cazorla , S. L. Y. Chang , Y. Yin , Q. Zhang , S. Lim , Y. Yao , R. Tian , Y. Wang , T. Zaman , H. Arandiyan , G. G. Andersson , J. Scott , P. Koshy , D. Wang , C. C. Sorrell , ACS Sustain. Chem. Eng. 2023, 11, 3370.

[86] L. Li , Y. Ma , G. Chen , J. Wang , C. Wang , Scr. Mater. 2023, 206, 2022.

[87] X. Jin , X. Li , L. Dong , B. Zhang , D. Liu , S. Hou , Y. Zhang , F.‐M. Zhang , B. Song , Nano Energy 2024, 123, 109341.

[88] X.‐L. Wang , Y. Xiao , Z.‐J. Lv , H. Yu , Y. Yang , X.‐T. Dong , J. Alloys Compd. 2020, 835, 155409.

[89] L. Wang , J. Zhao , X. Tang , S. Kuang , L. Qin , H. Lin , Q. Li , Surfaces and Interfaces 2023, 39, 102956.

[90] T. Naz , A. Rasheed , S. Ajmal , N. Sarwar , T. Rasheed , M. M. Baig , M. S. Zafar , D. J. Kang , G. Dastgeer , Ceram. Int. 2021, 47, 33454.

[91] N.‐N.‐T. Dang , V.‐A. Nguyen , N.‐D.‐T. Huynh , M.‐T. K. N. Van Hoang Luan , T.‐H. Tseng , S. Sagadevan , Y.‐H. Chang , M.‐V. Le , J. Water Process Eng. 2025, 71, 107196.

[92] D. Fernandes , C. W. Raubach , P. L. G. Jardim , M. L. Moreira , S. S. Cava , Ceram. Int. 2021, 47, 10185.

[93] T. Kuru , G. Yanalak , A. Sarilmaz , E. Aslan , A. Keles , M. T. Genc , F. Ozel , I. H. Patir , M. Kus , M. Ersoz , J. Photochem. Photobiol. A, Chemistry 2021, 436, 2023.

[94] B. Zhang , X. Jin , X. Li , L. Dong , D. Liu , Y. Zhang , Z. Zhao , Q. Ge , F.‐M. Zhang , Int. J. Hydrogen Energy 2024, 84, 480.

[95] A. Zhang , Z. Liu , B. Xie , J. Lu , K. Guo , S. Ke , L. Shu , H. Fan , Appl. Catal., B 2024, 279, 2020.

[96] W. Shen , N. Li , S. Zuo , M. Wu , G. Sun , Q. Li , M. Shi , J. Ma , Ceram. Int. 2022, 48, 15899.

[97] C. Porwal , S. Verma , M. Kumar , V. Singh Chauhan , R. Vaish , Nano‐Structures & Nano‐Objects 2023, 34, 100969.

[98] Q. Pan , J. Wang , H. Chen , P. Yin , Q. Cheng , Z. Xiao , Y. Zhao , H.‐B. Liu , J. Water Process Eng. 2023, 56, 104330.

[99] S. Li , Z. Liu , Z. Qu , C. Piao , J. Liu , D. Xu , X. Li , J. Wang , Y. Song , J. Photochem. Photobiol. A, Chem. 2023, 389, 2020.

[100] N. Li , B. Zhu , L. Huang , L. Huo , Q. Dong , J. Ma , Inorg. Chem. 2024, 63, 10011. 38752554 10.1021/acs.inorgchem.4c01213

[101] X. Huang , Z. B. Fang , W. Feng , Q. Tian , Z. Li , P. Liu , Dalton Trans. 2023, 52, 6097. 37063088 10.1039/d3dt00707c

[102] J. Hu , Y. Chen , Y. Zhou , L. Zeng , Y. Huang , S. Lan , M. Zhu , Appl. Catal., B 2023, 311, 2022.

[103] S.‐L. Guo , S.‐N. Lai , J. M. Wu , ACS Nano 2021, 15, 16106. 34543011 10.1021/acsnano.1c04774

[104] M. Guo , J. Zhong , W. Li , H. Hou , C. R. Bowen , X. Zhan , H. Yang , M. Yang , Z. Chen , D. Chen , Z. Liang , W. Yang , Nano Energy 2021, 127, 2024.

[105] S. Du , G. Li , X. Lin , S. Zhang , H. Xu , P. Fang , Chem. Eng. J. 2021, 409, 128157.

[106] Q. Han , Z. Han , Y. Wang , S. Zhang , J. Fang , H. Li , P. Fang , J. Colloid Interface Sci. 2023, 630, 460. 36334483 10.1016/j.jcis.2022.10.120

[107] H. Wei , F. Meng , H. Zhang , W. Yu , J. Li , S. Yao , Chem. Eng. J. 2024, 479, 147738.

[108] B. Kalaidhasan , L. Murugan , C. Jeyabharathi , R. Malini , S. Vengatesan , S. Vasudevan , S. Ravichandran , Carbon Lett. 2024, 34, 2259.

[109] R. M. N. Kalla , T. Kaliraja , S. K. Lakkaboyana , S.‐C. Kim , I. Kim , Carbon Lett. 2024, 34, 1229.

[110] R. Liu , X. Zhang , X. Han , Y. Sun , S. Jin , R.‐J. Liu , Carbon Lett. 2024, 34, 75.

[111] C. Medjili , N. Lakhdari , D. Lakhdari , A. Berchi , N. Osmani , I. Laourari , Y. Vasseghian , M. Berkani , Chemosphere 2023, 313, 137427. 36455660 10.1016/j.chemosphere.2022.137427

[112] X. Xu , Y. Zhao , Q. Yuan , Y. Wu , J. He , M. Fan , Carbon Lett. 2024, 34, 1629.

[113] N. R. Kim , J.‐H. Wee , C. H. Kim , D. Y. Kim , K. Kaneko , C.‐M. Yang , Carbon Lett. 2024, 34, 2317.

[114] Y. Vasseghian , M. Berkani , F. Almomani , E. N. Dragoi , Chemosphere 2021, 270, 129449. 33418218 10.1016/j.chemosphere.2020.129449

[115] D. Liu , J. Zhang , L. Tan , C. Jin , M. Li , B. Chen , G. Zhang , Y. Zhang , F. Wang , J. Colloid Interface Sci. 2023, 646, 159. 37187049 10.1016/j.jcis.2023.05.040

[116] J. Wang , C. Hu , Y. Zhang , H. Huang , Chin. J. Catal. 2022, 43, 1277.

[117] S. Lu , S. Zhang , L. Li , C. Liu , Z. Li , D. Luo , Chem. Eng. J. 2024, 483, 149058.

[118] P. T. T. Phuong , D.‐V. N. Vo , N. P. H. Duy , H. Pearce , Z. M. Tsikriteas , E. Roake , C. Bowen , H. Khanbareh , Nano Energy 2022, 95, 107032.

[119] M. Ebrahimi Farshchi , K. Asgharizadeh , H. Jalili , S. Nejatbakhsh , B. Azimi , H. Aghdasinia , M. Mohammadpourfard , J. Environ. Chem. Eng. 2022, 12, 2024.

[120] C. Hu , H.‐Y. Sun , X.‐M. Jia , H.‐L. Lin , J. Cao , S.‐F. Chen , ChemPhotoChem 2022, 6, 202200150.

[121] X. Wang , J. Jiang , L. Yang , Q. An , Q. Xu , Y. Yang , H. Guo , Appl. Catal., B 2022, 340, 2024.

[122] F. Peng , Z. Xie , H. Li , X. Kai , W. Wang , C. Wu , Catal. Lett. 2024, 154, 5271.

[123] Y. Lu , Y. Dong , W. Liu , Q. Jin , H. Lin , Chem. Eng. J. 2025, 508, 160935.

[124] D. Ding , Z. Li , S. Yu , B. Yang , Y. Yin , L. Zan , N. V. Myung , Sci. Total Environ. 2022, 824, 153790. 35150683 10.1016/j.scitotenv.2022.153790

[125] S. Deka , M. B. Devi , M. R. Khan , A. V. Keerthana , B. Choudhury , ACS Appl. Nano Mater. 2022, 5, 10724.

[126] S. Jia , Y. Su , B. Zhang , Z. Zhao , S. Li , Y. Zhang , P. Li , M. Xu , R. Ren , Nanoscale 2019, 11, 7690. 30946396 10.1039/c9nr00246d

[127] C. Sun , F. Li , J. Ren , J. Wu , G. Wang , L. Chen , Appl. Surf. Sci. 2021, 569, 151072.

[128] C. Fu Tan , W. L. Ong , G. W. Ho , ACS Nano 2015, 9, 7661. 26122026 10.1021/acsnano.5b03075

[129] P. Gotipamul , R. Maheswaran , S. Pandiaraj , S. Abdullah Alqarni , S. Chidambaram , Mater. Today Sustaina. 2015, 24, 2023.

[130] F. Bi , Z. Zheng , R. Li , R. Du , L. Zhao , S. Xiao , L. Wang , Chem. Eng. J. 2025, 507, 160781.

[131] Y. Cheng , T. Yu , X. Lei , B. Wang , W. Mu , X. Liu , R. Guo , Chem. Eng. J. 2025, 504, 158900.

[132] R. Chen , W. Gan , J. Guo , S. Ding , R. Liu , Z. Zhao , K. Yan , Z. Zhou , M. Zhang , Z. Sun , Chem. Eng. J. 2025, 502, 2024.

[133] G. Liu , H. Fei , J. Zhang , J. Wu , Z. Feng , S. Yang , F. Li , Y. Zhang , Chem. Eng. J. 2024, 493, 152596.

[134] N. H. Ly , L. Gnanasekaran , T. M. Aminabhavi , Y. Vasseghian , Curr. Opin. Chem. Eng. 2025, 47, 101087.

[135] Y. Ju , H. Lin , C. Chen , R. Hou , Z. Wang , T. Hao , M. Xu , Y. Tang , F. Chen , Chem. Eng. J. 2024, 498, 155790.

[136] H. Lv , Y. Liu , J. Zhou , Y. Bai , H. Shi , B. Yue , S. Shen , D.‐G. Yu , Chem. Eng. J. 2024, 484, 149514.

[137] D. Gautam , L. Saya , G. Gambhir , S. Hooda , Chem. Eng. J. 2024, 498, 154987.

[138] Y. Li , X. Zhang , R. Sha , T. Li , C. Hu , S. Tu , F. Chen , H. Huang , Chem. Eng. J. 2024, 480, 147976.

[139] C. Wang , Y. Jia , H. Zhou , M. Yang , J. Wang , X. Sun , C. Zhai , H. Zhang , L. Zhao , Chem. Eng. J. 2025, 506, 159263.

[140] Y. Li , C. Wang , M. Fan , X. Yu , G. Qin , L. Qiu , K. Yin , L. Wang , Chem. Eng. J. 2024, 498, 155584.

[141] X. Chen , H. Xu , C. Liu , Z. Wang , R. Wang , J. Wang , R. Pan , J. Qi , Y. Wang , Chem. Eng. J. 2024, 498, 155591.

[142] C. Zhao , C. Wang , X. Ren , S. Yuan , L. Zhao , L. Zhuang , B. Teng , Y. Wu , Chem. Eng. J. 2024, 498, 155202.

[143] J. Niu , T. He , C. Qiu , Z. Song , Chem. Eng. J. 2025, 508, 161206.

[144] T. P. A. T. Van Duc Bui , T. H. Vu , T. P. Dao , T. M. Aminabhavi , Y. Vasseghian , Appl. Catal. B, Environ. Energy 2025, 365, 2025.

[145] A. Verma , Y. P. Fu , Dalton Trans. 2024, 53, 4890. 38436475 10.1039/d4dt00081a

[146] Y. Zhu , H. Chen , L. Wang , L. Ye , H. Zhou , Q. Peng , H. Zhu , Y. Huang , Chin. Chem. Lett. 2024, 35, 108884.

[147] J. Jiang , L. Yu , J. Peng , W. Gong , W. Sun , Carbon Lett. 2025, 35, 417.

[148] Y. Vasseghian , E. N. Dragoi , F. Almomani , V. T. Le , Chemosphere 2022, 286, 131727. 34352554 10.1016/j.chemosphere.2021.131727

